# Identification of multiple hypoxia‐independent triggers of upper airway long‐term facilitation in a rat model of upper airway motor plasticity

**DOI:** 10.14814/phy2.70142

**Published:** 2025-03-11

**Authors:** Simon Lui, Arash Tadjalli, Jimmy Fraigne, John Peever

**Affiliations:** ^1^ Centre for Biological Timing and Cognition, Department of Cell & Systems Biology University of Toronto Toronto Ontario Canada; ^2^ College of Allopathic Medicine (NSU MD), Department of Medical Education Nova Southeastern University Fort Lauderdale Florida USA; ^3^ Department of Physiology University of Toronto Toronto Ontario Canada

**Keywords:** hypoglossal, hypoxia‐independent, locus coeruleus, long‐term facilitation

## Abstract

The respiratory control system can exhibit neuronal plasticity following exposures to repetitive respiratory challenges. For example, repeated obstructive apneas can trigger a form of respiratory plasticity that results in the enhancement of inspiratory hypoglossal (XII) motoneuron activity. This increase in respiratory motor output is known as hypoglossal long‐term facilitation (hLTF). In adult male Sprague–Dawley rats, we demonstrate that hLTF can also be triggered in the absence of repeated apneas by intermittent optogenetic stimulation of locus coeruleus (LC) neurons, or through pharmacological activation of adenosine‐A2a‐receptors at the level of brainstem XII motor pool. Both our pharmacological and optogenetic approaches that trigger hLTF require noradrenergic signaling through activation of α1‐noradrenergic receptors on hypoglossal motoneurons. We also use optical LC inhibition to reaffirm the importance of the LC in mediating apnea‐induced hLTF. These results demonstrate that hLTF can be triggered by multiple hypoxia‐independent stimuli, and for the first time, identify the LC as a key brainstem source for noradrenaline necessary for the expression of hLTF.

## 1 INTRODUCTION

The research presented in this paper addresses the neural circuits and mechanisms that underlie a form of plasticity that functions to augment upper airway respiratory motor output. In the last three decades, it has been well established that repeated episodes of hypoxia can trigger respiratory motor plasticity, termed long‐term facilitation (LTF) (Devinney et al., 2015; Tadjalli & Mitchell, 2019; Wilkerson et al., 2018). Long‐term facilitation in respiratory motor output manifests itself as a persistent increase (>1 h) in respiratory motor drive of important muscles of respiration, including the diaphragm (Braegelmann et al., 2019; Nichols & Mitchell, 2021; Tadjalli & Mitchell, 2019) and the genioglossus (Lui et al., 2018; Neverova et al., 2007; Tadjalli et al., 2010; Tadjalli & Mitchell, 2019)—the former traditionally referred to as phrenic long‐term facilitation (pLTF) and the latter referred to as hypoglossal long‐term facilitation (hLTF). The cellular mechanisms of intermittent‐hypoxia‐induced pLTF have been explored in great detail and the signaling cascades that orchestrate this form of plasticity are well defined. (Hoffman et al., 2012; Nichols & Mitchell, 2021; Tadjalli & Mitchell, 2019; Tahawi et al., 2001).

In our previous work, we discovered a distinct form of hypoxia‐independent hLTF. We found that repeated modulation of vagal feedback triggered by repeated obstructive apneas‐induced LTF of upper airway genioglossus muscle activity (hereafter referred to as hLTF) (Tadjalli et al., 2010). In our model, hLTF was triggered by vagal feedback and not by hypoxia because removal of vagal feedback even in the presence of episodic hypoxia prevented hLTF (Tadjalli et al., 2010). More importantly, we showed that activation of α1‐noradrenergic receptors at the hypoglossal motor pool is required for the expression of this form of respiratory motor plasticity (Tadjalli et al., 2010). While our previous work showed the need of endogenous noradrenergic receptor activation at the hypoglossal motor pool, the source of the relevant noradrenaline remained unknown. The aim of the present work was to identify this source and to further elucidate the cellular signaling mechanisms of this unique form of respiratory motor plasticity.

Here, we hypothesize that the locus coeruleus (LC) is potentially the main source of noradrenaline necessary for the expression of apnea‐induced hLTF. We had previously shown an increase in cFos expression within the LC following apnea‐induced hLTF expression, and that apnea‐induced hLTF can be abolished by inactivating LC neurons (Lui et al., 2018). Since vagus nerve manipulation causes changes in LC neuronal firing (Farrand et al., 2023), we hypothesize that the LC could be a key player in eliciting hLTF. Based on these lines of evidence, we proceeded to elucidate the role of the LC in mediating apnea‐induced hLTF.

By using combined pharmacological and optogenetic approaches, we show that intermittent optical stimulation of channelrhodopsin‐(ChR2) expressing LC neurons elicited hLTF, and it could be blocked via prior antagonism of α1‐noradrenergic receptors at the hypoglossal motor pool. We also show that optical inhibition of halorhodopsin‐(eNpHR) expressing LC neurons prevented apnea‐induced hLTF to reaffirm the importance of the LC in our model. In addition, we show that α1 noradrenergic‐receptor blockade at the hypoglossal motor pool *after* repeated apneas prevented hLTF development, demonstrating that apnea‐induced hLTF requires ongoing noradrenergic receptor signaling for its *maintenance* and not just initiation. Lastly, we show that Trk‐receptor inactivation at the hypoglossal motor pool prevented apnea‐induced hLTF and that pharmacological activation of Trk‐receptor signaling (via adenosine‐2a receptor signaling) at the hypoglossal motor pool was sufficient at inducing hLTF.

## 2 METHODS

### 2.1 Animals

Experiments were performed on anesthetized, spontaneously breathing adult male Sprague–Dawley rats. A total of 112 rats, aged 8–12 weeks, were included in this study. Rats were purchased from Charles River Laboratories (Wilmington, MA) and housed at the University of Toronto Cell and Systems Biology Animal Bioscience Facility. Rats were housed in pairs with unlimited access to food (LabDiet 5001; Arden Hills, MN) and water at room temperature on a 12‐h light–dark cycle (lights on at 7 am). A minimum of 1 week was given to allow animals to acclimatize to housing conditions before any procedures were performed. All experimental protocols were approved by the University of Toronto Animal Care Committee. All experimental procedures in this study were performed in accordance with both the Canadian Council on Animal Care and University of Toronto Animal Care Committee. All studies are reported in accordance with ARRIVE guidelines.

### 2.2 Surgical procedures

Anesthesia was introduced by placing rats into an induction chamber with 3.5% isoflurane in a 50/50 oxygen/nitrogen mix and maintained via a nose cone at 3% isoflurane. Rectal temperature was monitored and maintained at 37.5 ± 0.5°C via a servo‐controlled heating pad (09585; FHC, Bowdoinham, ME, or TC‐1000; CWE Inc.) throughout surgeries and experimental recordings. After complete absence of corneal and foot‐withdrawal reflexes, a tracheostomy was performed whereby a custom‐made silicone T‐tube cannula was inserted into the trachea just below the larynx. Anesthesia was maintained through the T‐tube for the remainder of the experiments at 2%–2.5% isoflurane set to a flow rate of 1 L per min. To prevent the accumulation of mucosal secretions that may occlude the tracheal T‐tube, a subcutaneous injection of atropine sulfate (0.4 mg/kg) was administered. The jugular vein was cannulated for administration of lactated Ringer's solution at a rate of 1.5 mL per hour. Two needle electrodes (F‐E2; Grass Technologies) were inserted into the genioglossus muscle, with one electrode on either side of the muscle to record EMG activity. To record diaphragm EMG activity, a 1–2 cm midline abdominal incision was made, and a custom‐made bipolar electrode was fastened onto the fascia of the right diaphragm. All incisions were closed with 9 mm wound clips (Becton Dickinson) to prevent tissue desiccation.

### 2.3 Stereotaxic injection of viral vector

Sterile stereotaxic surgery was performed to introduce an adeno‐associated virus (AAV) into the locus coeruleus (LC), or drug into the hypoglossal motor pool. Animals were placed in a stereotaxic setup (David Kopf Instruments) with their heads secured with ear bars and a snout clamp. Animals were draped and had their eyes covered with an ophthalmic ointment to prevent drying. Burr holes were drilled (TX Series, Foredom Electric Co.) at the surface of the skull to expose the dura, bilaterally above the LC at coordinates (relative to bregma) 10.0 mm posterior, 1.4 mm lateral, 7.5 mm ventral, or unilaterally above the hypoglossal motor pool (AP 14.5 mm, ML 0.2 mm, and DV 9.0 mm). Coordinates were guided by the stereotaxic brain atlas by Paxinos and Watson (1998). The dura was then punctured using a 25‐gauge sterile hypodermic needle. A stainless steel 28‐gauge cannula connected to a digital microinjection syringe pump (Pump 11 Elite; Harvard Apparatus) was then lowered to the target region to deliver 600–1000 nL of either AAV5‐hsyn‐ChR2(H134R)‐mCherry (4.1 × 10^12^ vg/mL), AAV5‐hsyn‐eNpHR3.0‐mCherry (6.7 × 10^12^ vg/mL), or AAV5‐hsyn‐mCherry (3.4 × 10^12^ vg/mL), purchased from the University of North Carolina Vectorcore. After microinjection, the incision was sutured, and postoperative care was given. Animals injected with a viral vector were given for 3–4 weeks for recovery and to allow for gene expression before experiments began.

### 2.4 Drug preparation and delivery

All drug solutions were made on the day of experiments. Terazosin (terazosin hydrochloride; Cat# T4680, 423.80 FW; Sigma‐Aldrich), an α1 noradrenergic receptor antagonist, was dissolved in lactated Ringer's then filtered (0.2 μm nylon; Thermo Fisher Scientific). Terazosin was delivered via reverse microdialysis (CMA‐11, membrane diameter 0.24 mm with 6 kDa cut‐off; Harbard Bioscience) at 1 μM perfusing at 2 μL per min. This concentration was chosen as it has been shown effective at antagonizing α1 noradrenergic receptors (Rouquier et al., 1994; Tadjalli et al., 2010). K252a (Cat# K1639, 467.47 FW; Sigma‐Aldrich, Canada), a tropomyosin related kinase (Trk) inhibitor, and CGS21680 (Cat# C141, CGS‐21680 hydrochloride hydrate; 536.98 FW; Sigma‐Aldrich, Canada), an adenosine A2a receptor agonist, were dissolved in 0.9% NaCl solution (saline) and delivered via reverse microdialysis. The microdialysis probe was lowered into the hypoglossal motor pool 20 min prior to any intervention. Drug delivery was controlled via a syringe pump driver and controller (Hive Syringe Pump Controllers, MD‐1020, BASi), and cannula placements were verified by postmortem histology.

### 2.5 Optogenetic manipulations

Optogenetic stimulation or inhibition was performed using 473 nm wavelength laser system (LRS‐0473, Laserglow) or a 532 nm wavelength laser system (LRS‐0532, Laserglow), respectively. Custom optic implants were made by connecting optic fiber to a ceramic ferrule (MM‐FER2007C‐2300, Precision Fibre Products) using epoxy. Only light output that was greater than 25 mW and less than 50 mW at the optic fiber tip, measured using a power meter (PM100A, ThorLabs), were used for experiments.

### 2.6 Electrophysiology recordings

Genioglossus and diaphragm EMG signals were amplified between 500 and 2000 Hz using a Super‐Z High Impedance Head Stage (cat# 10‐02010, CWE Inc.) and a BMA‐400AC/DC Bioamplifier (cat#09‐03010, CWE Inc.). Signals were filtered with a bandpass between 1 and 3000 Hz for EMG signals sampled at 1000 Hz. End‐tidal CO_2_ and temperature measurements were sampled at 40 Hz (Spike2 software, 1401 Interface; CED) and digitized (1 kHz; Micro1401; Cambridge Electronic Design). Integrated respiratory EMG activities were quantified using Spike2 software (Cambridge Electronic Design). All signals were stored on a computer for offline analysis.

### 2.7 Measurement of ET‐CO_2_ and O_2_ saturation

End‐tidal CO_2_ (ET‐CO_2_) was monitored in real‐time using a calibrated fast response CO_2_ analyzer (Model 17630; VacuMed or MicroCapster Endtidal CO_2_ analyzer, CWE Inc.) connected to the tracheal T‐tube. End‐tidal CO_2_ monitor was calibrated using carbagen (5% CO_2_ in 95% O_2_) before every experiment to measure percent CO_2_ during expiration. Arterial O_2_ saturation was estimated using a pulse oximeter designed for rodents, connected to the hind‐paw of the animal (MouseOx Pulse Oximeter; STARR Life Sciences Corp.) WINDAQ Waveform Browser software (Dataq Instruments) was used to digitize and analyze O_2_ saturation signals, which were then recorded using Spike2 software. Both variables were analyzed and quantified offline using Spike2 software.

### 2.8 Experimental protocol

All animals were divided into the following groups: Group 1: animals illuminated with intermittent 473 nm light after receiving the control virus (AAV5‐hsyn‐mCherry) (*n* = 6), Group 2: animals illuminated with intermittent 473 nm light receiving the excitatory virus (AAV5‐hsyn‐ChR2(H134R)‐mCherry) (*n* = 11), Group 3: animals illuminated with intermittent 473 nm light after receiving the excitatory virus but did not express hLTF due to off‐target probe tracts (*n* = 6), Group 4: animals illuminated with continuous 473 nm light after receiving the excitatory virus (*n* = 5) to elucidate underlying neural mechanisms, Group 5: animals illuminated with 473 nm light after receiving the excitatory virus plus terazosin perfusion at the hypoglossal motor pool (*n* = 8) to elucidate underlying neural mechanisms, Group 6: animals illuminated with 532 nm light after receiving the inhibitory virus (AAV5‐hsyn‐eNpHR3.0‐mCherry) (*n* = 9), Group 7: animals illuminated with 532 nm light after receiving the control virus (*n* = 10) and were paired with the inhibition group for analysis, Group 8: animals that received terazosin perfusion after repeated apnea intervention (*n* = 6), Group 9: animals that received saline perfusion alone (*n* = 7), Group 10: animals that received 1 μM terazosin perfusion alone (*n* = 6), Group 11: animals that received saline perfusion before repeated apneas (*n* = 7), Group 12: animals that received k252a perfusion (*n* = 13) before repeated apneas, Group 13: animals that received CGS21680 perfusion (*n* = 13), and finally Group 14: animals that received 1 μM terazosin perfusion after CGS21680 (*n* = 5). In all groups, after each experimental paradigm's surgical procedure, animals were left to stabilize for 30–60 min to establish a baseline activity for the genioglossus and diaphragm EMG muscle activity, ET‐CO_2_, and O_2_ saturation. After all experimental protocols, all activities were further recorded for 60 min. Control experiments of equal duration were also performed to account for potential time‐dependent fluctuations in physiological variables. At the end of each experiment, the animal was sacrificed with an overdose of isoflurane and brain tissue was collected for postmortem histology.

### 2.9 Objective 1—Can intermittent stimulation of the locus coeruleus elicit respiratory motor plasticity?

To determine whether intermittent optical stimulation of the LC can trigger hLTF, rats were infected with either AAV5‐hSyn‐ChR2(H134R)‐mCherry (*n* = 22) or AAV5‐hSyn‐mCherry (*n* = 6). Optic implants were inserted bilaterally into the LC and a baseline was established. In 11 animals, LC neurons were stimulated at 5 Hz for 15 s separated by 1‐min of no stimulation, repeated 10 times. This intervention mimics the time domain of repeated apnea protocol (Lui et al., 2018). The remaining animals were divided into three control groups: an off‐target control (*n* = 6) to determine the effect of stimulation outside the LC, a continuous stimulation control (*n* = 5) to determine the requirement for the intermittent nature of the stimulus, and an mCherry control (*n* = 6) with animals infected with a viral vector that is absent ChR2, to determine whether viral infection alone influenced the manifestation of hLTF.

### 2.10 Objective 2—Can optical silencing of locus coeruleus neurons prevent apnea‐induced hLTF?

To determine whether optical inactivation of the LC prevents apnea‐induced hLTF, rats were infected with either AAV5‐hSyn‐eNpHR3.0‐mCherry (*n* = 9) or AAV5‐hSyn‐mCherry (*n* = 10). Optic implants were inserted bilaterally into the LC and a baseline was established. LC neurons were then exposed to 532 nm light continuously while simultaneously delivering repeated obstructive apneas for 15 s separated by 1‐min recovery, repeated 10 times. A viral vector absent eNpHR was used to determine whether viral infection itself influenced the manifestation of hLTF.

### 2.11 Objective 3—Is the locos coeruleus the brainstem source for the relevant noradrenaline required for the induction of hypoglossal LTF?

The LC has been reported to co‐release multiple neurotransmitters (Fung et al., 1994; Trudeau, 2004). To determine whether hLTF elicited by intermittent LC stimulation requires noradrenergic signaling, separate group of rats (*n* = 8) infected with AAV5‐hSyn‐ChR2(H134R)‐mCherry received terazosin perfusion at the hypoglossal motor pool prior to optogenetic activation of hypoglossal motoneurons. Baseline activity was recorded for 60 min prior to intervention. Terazosin (1 μM) was perfused over the last 20 min at rate of 0.1 μL/min to antagonize α1‐noradrenergic receptors at the level of the hypoglossal motor pool prior to LC stimulation. LC neurons were then repeatedly stimulated with 473 nm light at 5 Hz for 15 s separated by 1 min of no stimulation, repeated 10 times.

### 2.12 Objective 4—Does apnea‐induced hLTF require α1‐noradrenergic receptor activation after hLTF induction?

In our previous study, we showed that apnea‐induced hLTF requires noradrenergic receptor signaling at the hypoglossal motor pool for its initiation. However, it is unknown if apnea‐induced hLTF required α1‐noradrenergic receptor activation *after* repeated apneas. To determine this we antagonized hypoglossal noradrenergic receptors after the repeated apnea protocol. Baseline activity was recorded for 60 min prior to the repeated apnea intervention (*n* = 6). In animals that exhibited LTF‐like increases in hypoglossal motor outflow at the 15‐min time point after apneas, terazosin (1 μM; *n* = 6) was perfused over 20 min at rate of 0.1 μL/min to antagonize α1‐noradrenergic receptors at the level of the hypoglossal motor pool. After 20 min, terazosin perfusion was switched back to saline solution for the remainder of the experiment. Results were compared to control animals (*n* = 7) that received repeated apneas plus saline perfusion at the hypoglossal pool. To account for the sole action of terazosin perfusion on hypoglossal motor activity, a separate group of animals were exposed to terazosin perfusion alone without exposures to repeated apneas (*n* = 6).

### 2.13 Objective 5—Does apnea‐induced hLTF require activation of Trk receptor signaling?

To determine whether apnea‐induced hLTF requires Trk receptor signaling, rats were perfused with k252a, a Trk receptor blocker, at a dose of 10 (*n* = 6) or 50 (*n* = 7) μM into the hypoglossal prior to the repeated apnea intervention. Saline perfusion was resumed after 20 min, and activity was recorded for 60 min after the repeated apnea protocol.

### 2.14 Objective 6—Can adenosine A2a receptor activation at the hypoglossal motor pool elicit hLTF?

To determine whether activation of adenosine A2a receptors on hypoglossal motoneurons can elicit hLTF, CGS21680 (an adenosine A2a receptor agonist) was perfused into the hypoglossal motor pool. A2a receptor activation influences the actions of neuromodulators such as neurotrophins, leading to an enhancement of excitatory synaptic drive via mechanisms that involve transactivation of neurotrophin receptors, including TrkB receptors (Golder et al., 2008; Lee & Chao, 2001). CGS21680 was perfused for 20 min at the hypoglossal motor pool at a dose of 25 (*n* = 7) or 50 (*n* = 6) μM before resuming saline perfusion. Results were compared to a separate set of time‐matched control animals that received saline perfusion alone (*n* = 7).

### 2.15 Objective 7—Does noradrenergic signaling influence the expression of adenosine‐2a‐receptor‐induced hypoglossal motor facilitation?

The aim of this experimental protocol was to determine whether adenosine‐2a‐receptor‐induced hLTF requires noradrenergic signaling. To investigate this, α1‐noradrenergic receptors were antagonized *after* induction of adenosine‐2a‐receptor‐induced hypoglossal motor facilitation (*n* = 5). Specifically, 15 min after adenosine‐A2a receptor activation, α1‐noradrenergic receptors were antagonized via terazosin perfusion over 20 min at a concentration of 1 μM. Saline perfusion was then resumed, and activity was further recorded for 60 min.

### 2.16 Data analysis

All animals that survived the surgical intervention and subsequent recording were included in this study. Peak integrated inspiratory genioglossus and diaphragm EMG amplitudes as well as respiratory frequency were quantified on a breath‐by‐breath basis in 60 s intervals during all experiments. Inspiratory amplitude, respiratory frequency, estimated arterial O_2_ saturation, and percent of end‐tidal CO_2_ levels were expressed as a percent change from baseline ± standard deviation (SD). Baseline values for inspiratory amplitude and respiratory frequency were acquired during the 240 s prior to each experimental intervention. Data were quantified and expressed before (i.e., baseline) and at 15, 30, 45, and 60 min after experimental interventions. Each animal was determined to have exhibited hLTF if they met two specific criteria: (1) genioglossus inspiratory amplitude was two standard deviations above baseline levels at the 60‐min time point after intervention; and (2) genioglossus inspiratory amplitude averaged over 60 min post‐intervention was two standard deviations above baseline levels. If an animal failed either criterion following an intervention, they were not considered to have exhibited hLTF and were placed in a separate group henceforth known as nonresponders. This allowed for a clear separation between animals that exhibited hLTF and nonresponders.

### 2.17 Histology

Immunohistochemical staining was performed on all rats to verify (1) probe tract locations, (2) cell activity as determined by c‐Fos expression, and (3) cell phenotype as determined by tyrosine hydroxylase staining to identify noradrenergic neurons. At the end of each experiment, rats were killed and perfused with 4% paraformaldehyde in 0.1 M phosphate buffer. Brains were extracted and stored in 4% paraformaldehyde overnight, followed by a cryoprotection step by submerging brains into 30% sucrose in 0.1 M PB solution until brains were saturated. Brains were then immersed in Tissue‐Tek OCT Compound (Electron Microscope Sciences) and frozen on dry ice. Frozen brains were then sliced in a cryostat (CM3050 S, Leica Microsystems) at 40 μm coronal sections. Immunohistochemistry was used to identify colocalized expression of c‐Fos and tyrosine hydroxylase. Primary antibody rabbit anti‐c‐Fos (1:5000 dilution, Immunostar, cat# 26209, lot# 113018B, RRID: AB_572267) was used in conjunction with mouse anti‐TH (1:1000 dilution, Immunostar, cat# 22941, lot# 907001, RRID: AB_572268). After 48 h of incubation at 4°C, biotinylated secondary antibodies, biotinylated goat anti‐rabbit IgG (1:800 dilution, Vector Laboratories, cat# BA‐1000, lot# Z0619, RRID: AB_2313606), and biotinylated goat anti‐mouse IgG (1:600 dilution, Vector Laboratories, cat# BA‐9200, lot# W2206, RRID: AB_2336171) were used. To visualize, an avidin biotin complex (ABC) kit (VECTASTAIN Elite ABC HRP Kit, Vector Laboratories, PK‐6100, RRID: AB_2336819) used in conjunction with a 3,3′‐diaminobenzidine (DAB) peroxidase kit (DAB Kit, Vector Laboratories, SK‐4100, RRID: AB_2336382) to oxidize DAB, providing a brown‐black color in the nuclei of c‐Fos positive cells, and NovaRed (NovaRed Kit, Vector Laboratories, SK‐4800, RRID: AB_2336845) was used to provide a contrasting red color to identify noradrenergic cells. The location of lesion tracts was plotted on standardized brain maps (Paxinos & Watson, 1998). In experiments where virally infected LC neurons expressed mCherry, tissue was incubated in primary rabbit anti‐mCherry (1:500 dilution, Novus Biologicals, cat# NBP2‐25157, lot# 12016, RRID: AB_2753204) and secondary goat anti‐rabbit Cy3 antibodies (1:500, Jackson ImmunoReserach Labs, cat# 111‐167‐003, lot#78034, RRID: AB_2313593). To identify noradrenergic cells, tissue was incubated again in primary mouse anti‐TH with secondary goat anti‐mouse Alexa Fluor 488 (1:500, Jackson ImmunoResearch Labs, cat# 111‐567‐003, lot# 130258, RRID: AB_2338058). Tissue was then counterstained with DAPI. Sections were imaged with the upright fluorescent and confocal microscope (AxioObserver Z1; Zeiss), or under brightfield.

### 2.18 Cell quantification

Sections were analyzed under upright fluorescent and confocal microscopes (AxioObserver Z1; Zeiss). Cells with unambiguous c‐Fos expression were manually counted with observers blinded to the treatment. A cell was considered c‐Fos+ if a cell expressed a black nucleus and excluded cells that expressed nuclei that were light/medium brown (which may or may not be c‐Fos+). This level of stringency ensured that we only identified c‐Fos+ cells and thereby excluded the possibility of identifying false positive cells. Regions of interest were identified using the rat brain atlas (Paxinos & Watson, 1998), and counted using ImageJ. Three representative slices were taken across the rostral/caudal axis for each region per animal, and this sampling strategy is based on a recent study that showed that the distribution of LC projections had no specific organization across anterior/posterior or medial/lateral axes (Schwarz et al., 2015). Each image was 0.5 × 0.5 mm and encompassed the structure of interest. Fluorescent staining was used to identify mCherry and tyrosine hydroxylase (TH) positive cells. In these studies, cells were manually counted for each image with the experimenter blinded to the treatment.

### 2.19 Statistical analysis

All datasets passed normality. Comparisons within a treatment across time were made using a one‐way repeated measure analysis of variance (one‐way RM ANOVA) and post hoc comparisons were performed using the Dunnett test. Comparisons between treatments for each respiratory variable were made using a two‐way RM ANOVA with post hoc Bonferroni test to infer statistical significance. To determine whether an intervention influenced hLTF expression, a chi‐square test was performed to determine whether an intervention significantly correlated with hLTF expression. In addition, we performed a Firth logistic regression to determine whether predictors (e.g., an intervention such as repeated apneas) influenced hLTF expression. This differs from a chi‐square test as a logistic regression can model whether an intervention can predict an outcome. All animals (i.e., hLTF‐expressing animals and nonresponders) are included to determine whether an intervention influenced hLTF expression. Lastly, comparisons of respiratory muscle activity between all groups were also made using an ordinary least square (OLS) linear regression. This approach allowed for comparisons of muscle activity in all animals (i.e., hLTF‐expressing animals and non‐responders), in all treatment groups (i.e., intervention and controls), at a specific time point to infer statistical significance. Cell counts between groups were compared using a one‐way ANOVA and post hoc comparisons were performed using the Bonferroni test, or a student *t*‐test where applicable. All statistical analyses used GraphPad Prism (Prism v5.0, GraphPad), STATA (v5.10), and R Studio (v3.4.3). Data are presented as a mean + standard deviation.

## 3 RESULTS

### 3.1 LC cells were equally infected by viral vectors across all groups

**FIGURE 1:**
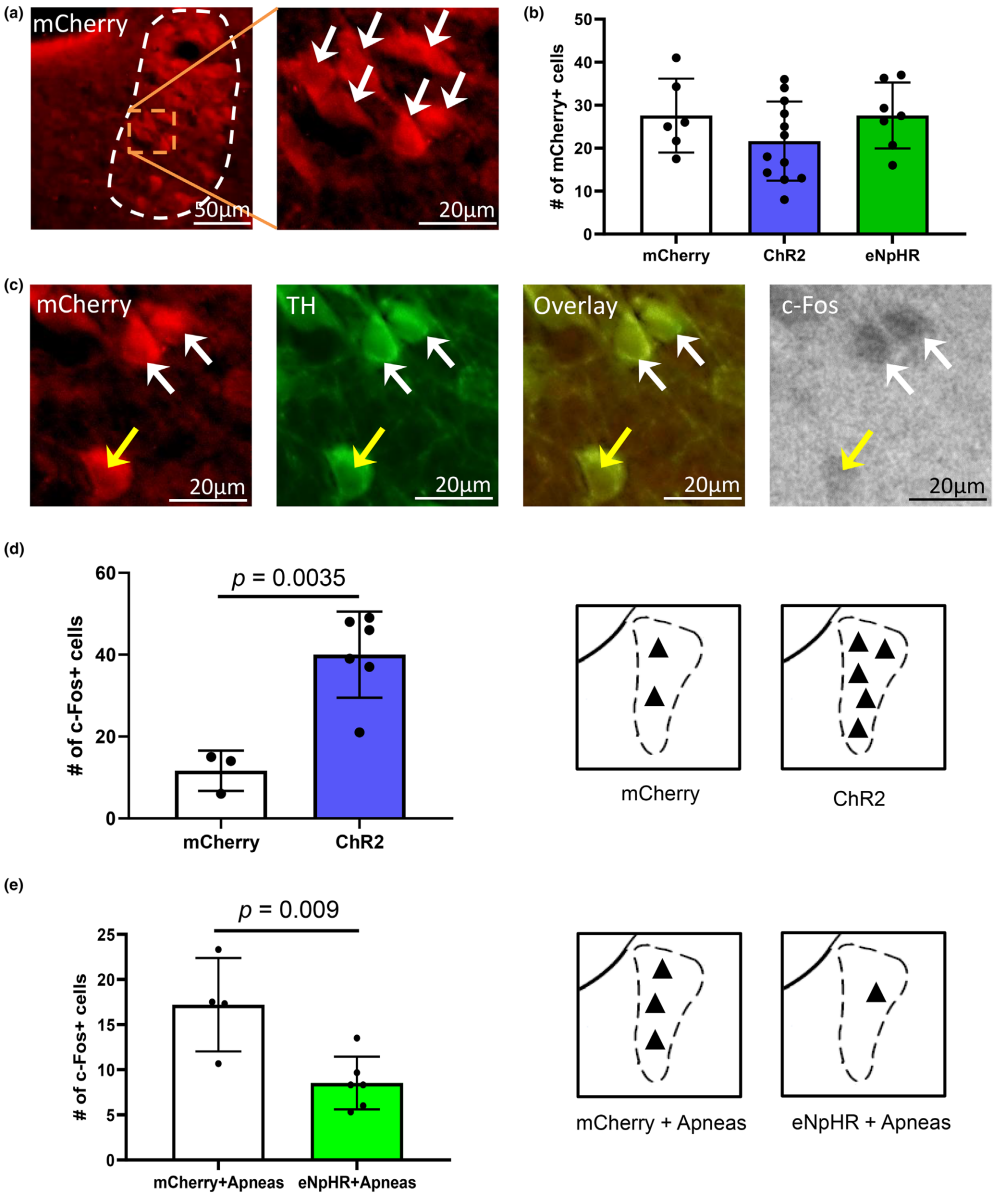
c‐Fos expression following light‐induced manipulation to ChR2‐ or eNpHR‐ expressing LC cells. Following microinjection of a viral construct AAV5‐hSyn‐ChR2(H134R)‐mCherry (*n* = 13), AAV5‐hSyn‐eNpHR3.0‐mCherry (*n* = 6), or AAV5‐hSyn‐mCherry (*n* = 6), expression of mCherry, tyrosine hydroxylase (TH), and c‐Fos was quantified with experimenters blinded to the treatment. All infected animals expressed an equal number of mCherry positive cells (a, b) (1‐way ANOVA, *F* = 1.153, *p* = 0.3333). (c) Histological example showing LC neurons that are mCherry positive (red) to identify virally infected cells, TH positive (green) to identify noradrenergic cells, the overlay (third panel), and c‐Fos (black) to identified activated cells. Visualization of c‐Fos was under bright field and therefore was not overlaid with fluorescent images in earlier panels. White arrows point to infected noradrenergic cells that were activated (i.e., c‐Fos positive). Yellow arrow points to infected noradrenergic cell that was considered c‐Fos negative (i.e., not activated). (d) Cells infected with the excitatory opsin, ChR2 (blue bar), displayed a significant increase in c‐Fos expression following intermittent light stimulation compared to animals absent any opsin (mCherry, white bar) (unpaired t‐test, LC Stim. vs. mCherry, *t*_(7)_=4.326, *p* = 0.0035). Approximate location of c‐Fos positive cells in the LC (dotted outline) are shown on the right for animals absent any opsin (mCherry) and animals expressing ChR2. (e) Cells infected with the inhibitory opsin, eNpHR (green bar), displayed a significant decrease in c‐Fos expression following light exposure and repeated obstructive apneas compared to animals absent any opsin (mCherry + Apneas, white bar) but received light exposure and repeated apneas (unpaired *t*‐test, LC inactivation + apneas vs. mCherry + apneas, *t*_(8)_ = 3.4282, *p* = 0.009). Approximate location of c‐Fos positive cells are shown on the right for animals absent any opsin (mCherry) and animals expressing eNpHR following repeated apneas. Data presented as mean + SD.

To confirm our viral injections yielded equal viral infection rates across animals that received the empty viral vector (AAV5‐hSyn‐mCherry), the viral vector containing the stimulating opsin, channelrhodopsin2 (ChR2) (AAV5‐hSyn‐ChR2(H134R)‐mCherry), or the inhibitory opsin, halorhodopsin (eNpHR) (AAV5‐hSyn‐eNpHR3.0‐mCherry). This is important as it addresses the quality of the viral vectors used across all treatment groups. No difference in mCherry expression across all groups was observed (1‐way ANOVA, *F* = 1.153, *p* = 0.3333 Figure 1a,b), suggesting viral infection rates were equal for all animals used in these studies. LC cells co‐expressing mCherry and tyrosine hydroxylase (TH) were quantified and we found 61 ± 14% of noradrenergic LC cells were infected.

To determine whether ChR2 was functional within LC neurons, we delivered light to infected LC neurons and found c‐Fos expression in LC cells was increased by 49 ± 13% compared to animals absent ChR2 (unpaired *t*‐test, LC Stim. vs. mCherry, *t*_(7)_=4.326, *p* = 0.0035, Figure 1c,d). To determine whether eNpHR was functional within LC neurons, we delivered light to infected LC neurons while simultaneously attempting to activate LC cells using apnea‐induced hLTF. Following this intervention, we observed fewer c‐Fos positive neurons in the LC compared to animals absent eNpHR and exhibited hLTF (unpaired *t*‐test, LC inactivation + apneas vs. mCherry + apneas, *t*_(8)_=3.4282, *p* = 0.009, Figure 1e). This suggests that the virally‐expressed opsins are functioning as intended.

### 3.2 Baseline genioglossus activity decreased during optical inactivation of eNpHR‐expressing LC neurons

**FIGURE 2:**
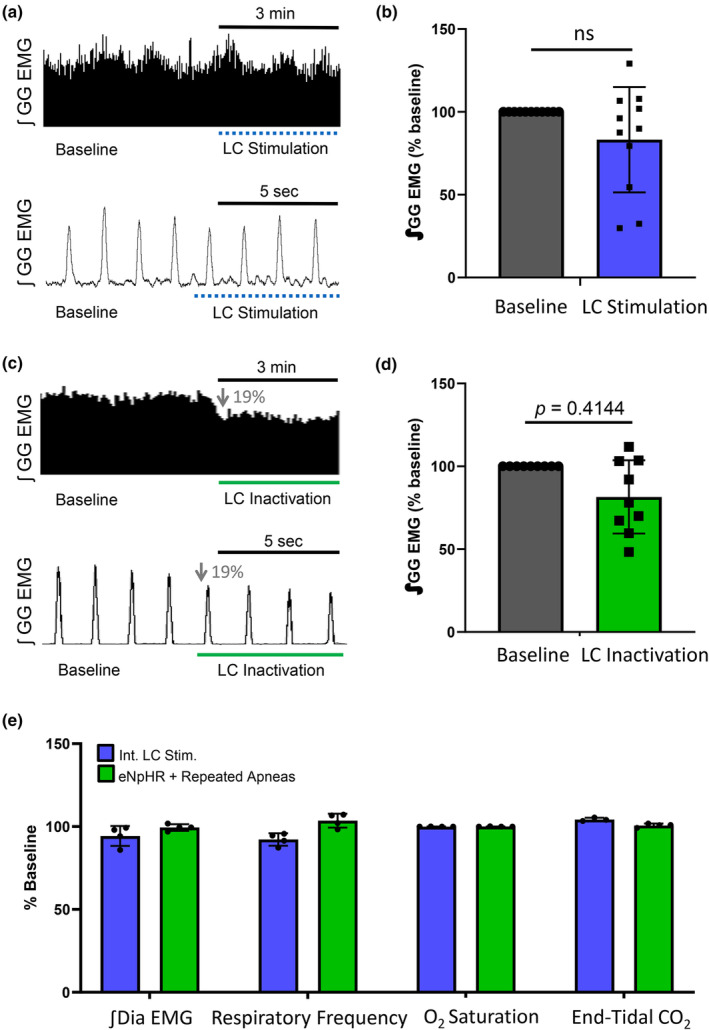
Genioglossus motor output decreased during optical LC inactivation. (a) Raw trace of integrated genioglossus amplitude (∫GG EMG) recorded from an anesthetized spontaneously breathing rat, before (baseline) and during LC stimulation (dotted blue line). Bottom panel depicts a higher temporal resolution of genioglossus amplitude during the switch from light off to light on. (b) Group data (*n* = 11) comparing genioglossus amplitude at baseline (black bar) and during intermittent LC stimulation (blue bar), where no significant difference was observed (paired *t*‐test, *t*_(8)_ = 0.8608, *p* = 0.4144). (c) Raw trace showing genioglossus activity before (baseline) and during LC inactivation (green line). Bottom panel shows higher temporal resolution genioglossus activity before and after the laser was turned on and the subsequent decrease in genioglossus amplitude. (d) In the group data (*n* = 9), baseline genioglossus amplitude (black bar) was significantly decreased following LC inactivation (green bar) by 19 ± 7% from baseline (paired *t*‐test, *t*_(8)_ = 2.506, *p* = 0.0366). (e) In both groups (LC activation or inactivation), integrated diaphragm amplitude, respiratory frequency, oxygen saturation levels, and end‐tidal CO_2_ levels were unaffected (paired *t*‐test, diaphragm: *t*_(3)_ = 1.511, *p* = 0.2279, respiratory frequency: *t*_(3)_ = 1.965, *p* = 0.1442, O_2_ saturation: *t*_(3)_ = 0.1060, *p* = 0.9223, end‐tidal CO2: *t*_(2)_ = 0.3183, *p* = 0.7804). Data presented as mean + SD.

While determining whether virally‐expressed eNpHR was functional, we observed a decrease in baseline genioglossus activity during optical LC inhibition. When LC neurons were exposed to intermittent light stimulation, we found no change in genioglossus amplitude (Figure 2a,b), or any other respiratory variable measured (paired *t*‐test, genioglossus: *t*_(8)_=0.8608, *p* = 0.4144, diaphragm: *t*_(3)_=1.511, *p* = 0.2279, respiratory frequency: *t*_(3)_=1.965, *p* = 0.1442, O_2_ saturation: *t*_(3)_=0.1060, *p* = 0.9223, end‐tidal CO_2_: *t*_(2)_=0.3183, *p* = 0.7804. Figure 2e). However, when eNpHR‐expressing LC neurons were inactivated with a continuous pulse of light, we found genioglossus amplitude decreased by 19 ± 7% (paired *t*‐test, *t*_(8)_=2.506, *p* = 0.0366, Figure 2c,d), but no change was observed in any other respiratory variable measured (paired *t*‐test, diaphragm: *t*_(3)_=0.5763, *p* = 0.6048, respiratory frequency: *t*_(3)_=1.708, *p* = 0.1862, O_2_ saturation: *t*_(3)_=0.2496, *p* = 0.8190, end‐tidal CO_2_: *t*_(3)_=0.3068, *p* = 0.3068. Figure 2e). The decrease in genioglossus motor output following optical inhibition of eNpHR‐expressing LC neurons suggests that LC neurons provide an endogenous excitatory drive to hypoglossal motoneurons.

### 3.3 Intermittent LC activation triggers elicits respiratory motor plasticity

**FIGURE 3:**
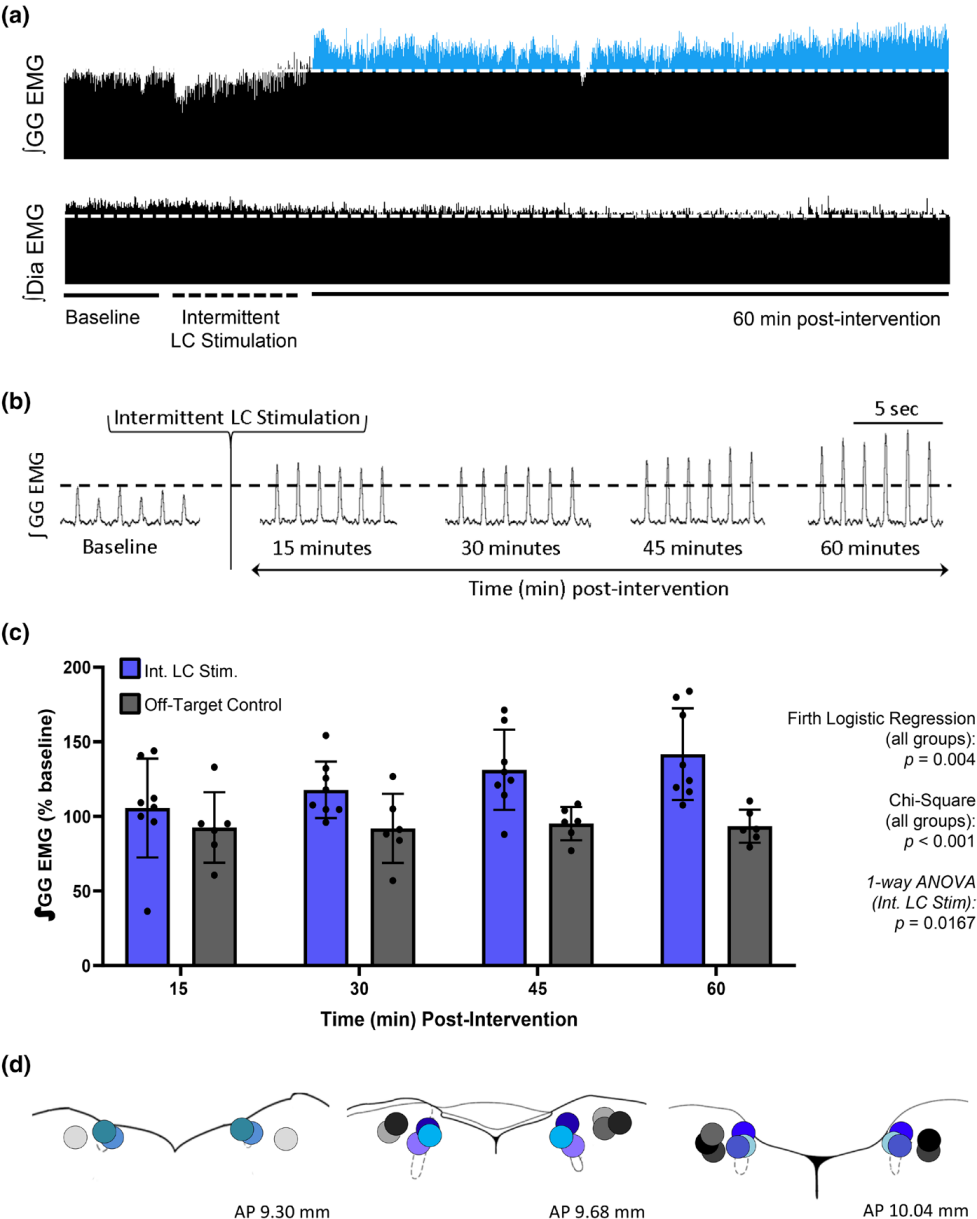
LTF of genioglossus motor activity is elicited after intermittent optical stimulation of the LC neurons. (a) Integrated inspiratory genioglossus motor output (∫GG EMG) recorded from an anesthetized spontaneously breathing rat, depicting baseline genioglossus amplitude (dotted line) and the subsequent increase in EMG amplitude after intermittent LC stimulation (i.e., LTF). (b) High‐temporal resolution EMG traces showing genioglossus activity at baseline, 15, 30, 45, and 60 min after intermittent LC stimulation. (c) Group data showing an increase in inspiratory genioglossus activity following intermittent LC stimulation (*n* = 8, blue bars) compared to animals given the same intervention but outside of the LC nuclei (i.e., Off‐Target Stimulation, *n* = 6, black bars) at 15, 30, 45, and 60 min after LC stimulation. Intermittent LC stimulation induced an increase in inspiratory genioglossus amplitude of 41 ± 10% at 60 min compared to baseline (1‐way RM ANOVA, *F* = 3.629, *p* = 0.0167), suggesting LC stimulation alone can trigger LTF. When compared to control groups (Off‐Target Control, mCherry Control, and Continuous LC Stimulation), we ran a Firth Logistic Regression and a chi‐square test with all groups, including nonresponders, and we found intermittent LC stimulation significantly correlated with hLTF expression (Firth logistic regression, Int. LC stimulation vs. controls, *p* = 0.004; chi‐square test, Int. LC Stimulation vs. controls, Chi2 = 10.8107, *p* < 0.001). (d) Optic probe tract locations for animals that received on‐target LC stimulation (blue circles), or off‐target stimulation (gray circles). Dotted outline represents the LC. Each shade represents an individual animal. Data presented as mean + SD.

We tested the hypothesis that the activation of the LC could trigger hLTF by modeling a stimulation protocol that is similar to the repeated obstructive apneas trigger. By optically stimulating LC neurons in the same pattern (i.e., stimulation at 5 Hz for 15 s separated by 1 min of no stimulation, repeated 10 times), repeated optical stimulation of ChR2‐expressing LC neurons increased genioglossus amplitude by 41 ± 10% at 60 min poststimulation (1‐way RM ANOVA, *F* = 3.629, *p* = 0.0167, Figure 3a–c), suggesting intermittent LC stimulation can trigger hLTF.

The viral vector used in this study was nonspecific and therefore infected all neurons. To address the nonspecific nature of the viral vector used, we stimulated cells 0.2–0.4 mm lateral to the LC in an identical fashion, whereupon we found no change in genioglossus activity (1‐way RM ANOVA, *F* = 3.898, *p* = 0.8289, Figure 3c), or any effect on other respiratory variables measured (2‐way RM ANOVA, diaphragm: *F* = 0.5053, *p* = 0.7320, respiratory frequency: *F* = 1.329, *p* = 0.2726, O_2_ saturation: *F* = 0.5756, *p* = 0.6820, Figure S1). This suggests that the manifestation of hLTF was due to the stimulation of LC cells, and not the cells near the LC.

To determine whether intermittent LC stimulation was significantly more likely to induce hLTF expression compared to controls, we performed a chi‐square test and a Firth logistic regression to model the probability of hLTF expression. We included all animals that received intermittent LC stimulation and all control groups (Off‐Target Stimulation, mCherry controls, continuous stimulation control). By including all animals regardless of whether they expressed hLTF following our intervention, we found intermittent LC stimulation significantly correlated with hLTF expression (chi‐square test, Int. LC Stimulation vs. controls, χ^2^ = 10.8107, *p* < 0.001; Firth logistic regression, Int. LC stimulation vs. controls, *p* = 0.004). Taken together, this suggests that intermittent LC stimulation is significantly more likely to induce hLTF compared to controls.

### 3.4 hLTF requires a minimum threshold activation of LC cells

It is important to note that LTF does not always manifest following a trigger. In studies of long‐term potentiation (LTP), plasticity is elicited 50%–90% of the time following a trigger (Abraham et al., 1993; Watanabe et al., 2002). In LTF studies, the percentage of nonresponders is typically not reported. Our next goal was instead aimed to examine why hLTF was not expressed in these animals. One possibility could be due to fewer infected noradrenergic LC neurons. Therefore, we quantified the total number of double‐labeled mCherry and tyrosine hydroxylase (TH) positive cells in animals that did and did not exhibit hLTF following LC stimulation. What we found was that the number of cells expressing both mCherry and TH were not significantly different between animals that exhibited hLTF and those that did not (unpaired *t*‐test, ChR2 LTF vs. ChR2 No LTF, *t*_(7)_=1.086, *p* = 0.3134; Figure S2a). This suggests that an unequal infection of noradrenergic LC cells was not the reason for a lack of LTF observed following intermittent LC stimulation in these animals.

Next, we examined the c‐Fos expression in noradrenergic LC cells of animals that exhibited hLTF and in nonresponders. More double‐labeled c‐Fos and TH positive cells in animals that exhibited hLTF were observed compared to non‐responders (unpaired *t*‐test, Int.LC Stim. LTF vs. Int.LC Stim. No LTF, *t*_(7)_=2.499, *p* = 0.0411. Figure S2b). This suggests the lack of hLTF in nonresponders may be a result of insufficient LC stimulation. This is consistent with previous work that showed higher c‐Fos expression in animals that exhibited hLTF compared nonresponders (Lui et al., 2018). In addition, this is further supported by studies that showed noradrenergic LC neurons naturally fire synchronously when sufficiently stimulated (Aston‐Jones & Bloom, 1981; Christie et al., 1989; Ishimatsu & Williams, 1996). It is possible that in some animals, intermittent LC stimulation did not meet the threshold required to trigger a sufficient response in LC activity and therefore could not elicit hLTF.

### 3.5 Intermittent light exposure on mCherry‐expressing LC cells does not elicit respiratory motor plasticity

**FIGURE 4:**
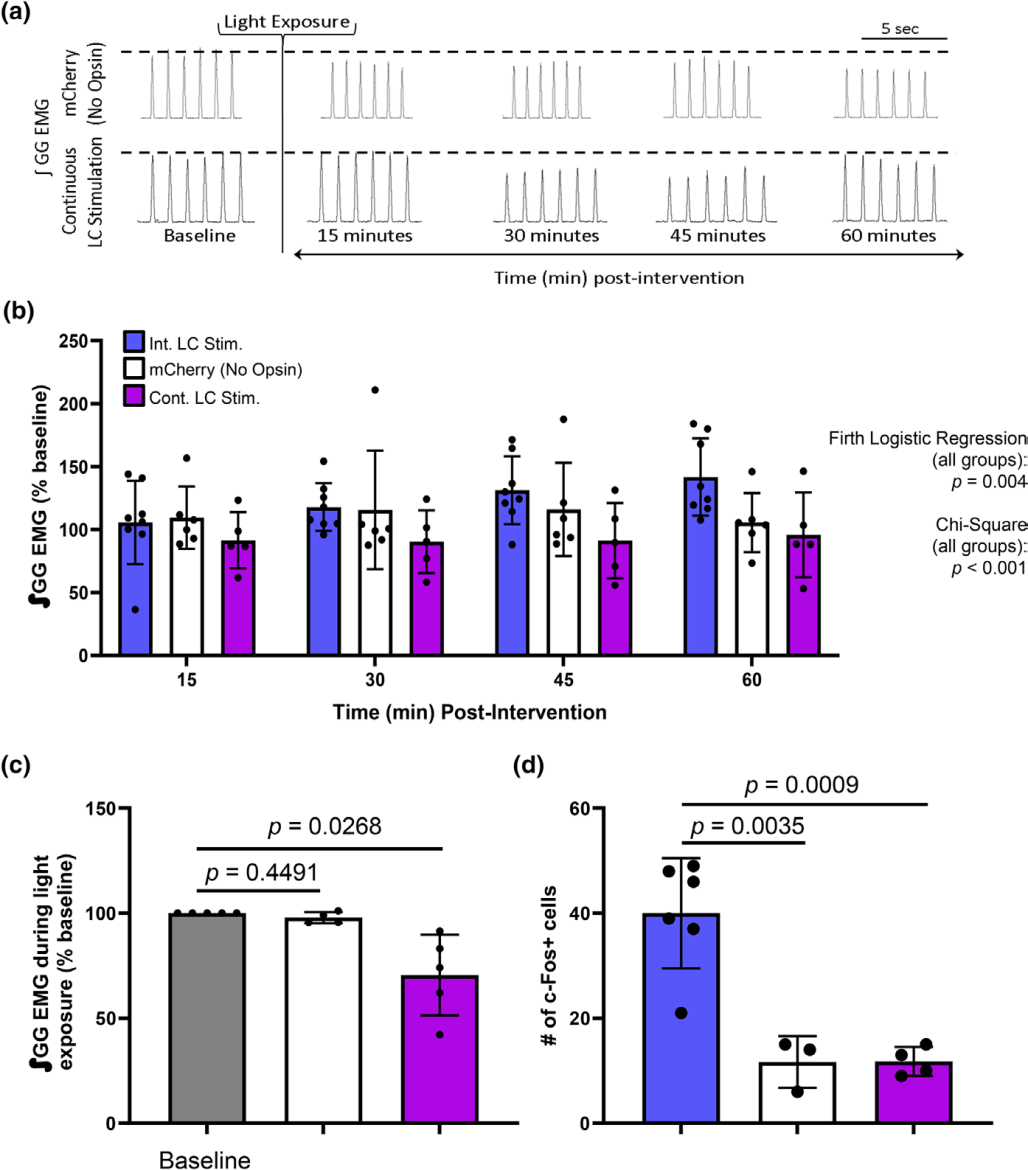
Intermittent LC stimulation activates ChR2‐expressing LC neurons more than non‐ChR2‐expressing mCherry neurons or continuous LC stimulation. (a) Representative EMG traces of integrated inspiratory genioglossus activity recorded from an anesthetized spontaneously breathing rat after intermittent light pulses in an animal with no opsin (i.e., mCherry, top trace) or continuous light exposure in ChR2‐expressing LC cells (i.e., Continuous LC Stimulation, bottom trace). Dotted line represents baseline activity. (b) Group data showing no change in inspiratory genioglossus activity in mCherry (1‐way RM ANOVA, mCherry, *F* = 0.7763, *p* = 0.5535) and continuous LC stimulation (1‐way RM ANOVA, *F* = 0.4031, *p* = 0.8037) groups at 15, 30, 45, and 60 min compared to ChR2‐expressing animals that received intermittent LC stimulation (1‐way RM ANOVA, *F* = 3.629, *p* = 0.0167). As shown earlier, when these groups are compared to intermittent LC stimulation, we found intermittent LC stimulation significantly correlated with hLTF expression (Firth logistic regression, Int. LC stimulation vs. controls, *p* = 0.004; chi‐square test, Int. LC Stimulation vs. controls, χ^2^ = 10.8107, *p* < 0.001). (c) Group data showing integrated genioglossus activity (∫GG EMG) decreased during continuous light exposure (purple bars, paired *t*‐test, genioglossus at baseline vs. during continuous stimulation: *t*_(4)_ = 3.418, *p* = 0.0268), but had no effect in animals with no opsins (i.e., mCherry, white bars, paired *t*‐test, genioglossus at baseline vs. during mCherry stimulation: *t*_(3)_ = 0.8683, *p* = 0.4491) relative to percent baseline (black bars). (d) Group data showing more c‐Fos positive cells in ChR2‐expressing animals that received intermittent LC stimulation (blue bars) and exhibited LTF compared to mCherry (white bars, unpaired *t*‐test, Intermittent stimulation vs. mCherry, *t*_(7)_ = 4.326, *p* = 0.0035) or animals given continuous LC stimulation (purple bars, *n* = 4, unpaired *t*‐test, intermittent stimulation vs. continuous stimulation, *t*_(8)_ = 5.163, *p* = 0.0009). Data presented as mean + SD.

Recent reports have suggested that light exposure in naïve mice (i.e., mice absent any opsin) can cause heat‐induced cell damage (Qian & Gu, 2005), heat‐induced cell firing (Reig et al., 2010; Stujenske et al., 2015), and photodilation of blood vessels (Rungta et al., 2017) To control for the potential adverse effects caused by light exposure on hLTF expression, we performed an identical stimulation protocol in animals infected with an empty viral vector (i.e., AAV5‐hSyn‐mCherry). Optical exposure of mCherry‐expressing LC neurons did not influence genioglossus activity (1‐way RM ANOVA, mCherry, *F* = 0.7763, *p* = 0.5535, Figure 4a,b), nor diaphragm activity, respiratory frequency, and blood oxygen saturation (Figure S3). These data indicate that light per se is not responsible for hLTF, but it is the intermittent activation of LC neurons themselves.

### 3.6 An intermittent, but not continuous, pattern of LC stimulation is required for LTF expression

Repeated episodes of hypoxia have been shown to be required for LTF expression (Baker & Mitchell, 2000). This intermittent nature was again demonstrated when intermittent cooling of vagal afferents triggered LTF, but a single cooling episode of equal duration did not (Tadjalli, 2012). We wanted to determine whether the intermittent nature of optical stimulation was required to trigger LTF. Here, we show that continuous light exposure to ChR2‐expressing LC cells did not trigger hLTF (1‐way RM ANOVA, *F* = 0.4031, *p* = 0.8037. Figure 4b). We again did not observe any change in other respiratory variables measured over time (Figure S3), suggesting continuous LC stimulation had minimal effect on respiratory output.

During continuous optical stimulation, we observed a decrease in genioglossus activity to 70 ± 19% of baseline levels (paired *t*‐test, genioglossus at baseline vs. during continuous stimulation: *t*_(4)_=3.418, *p* = 0.0268. Figure 4c). The decrease in genioglossus motor output may suggest that ChR2‐expressing LC cells exposed to continuous light experienced depolarization block, effectively preventing cell activation. This is supported in studies that used prolonged LC stimulation to induce depolarization block (Adams & Foote, 1988), and is further supported by histological c‐Fos expression. Specifically, continuous LC stimulation had fewer activated cells (i.e., c‐Fos positive cells) than intermittent LC stimulation (unpaired *t*‐test, Int. LC Stim. vs. Continuous LC Stim., *t*_(8)_=5.163, *p* = 0.0009. Figure 4d), suggesting LC cells were less activated following continuous stimulation.

### 3.7 Optical inactivation of the LC abolished apnea‐induced hLTF

**FIGURE 5:**
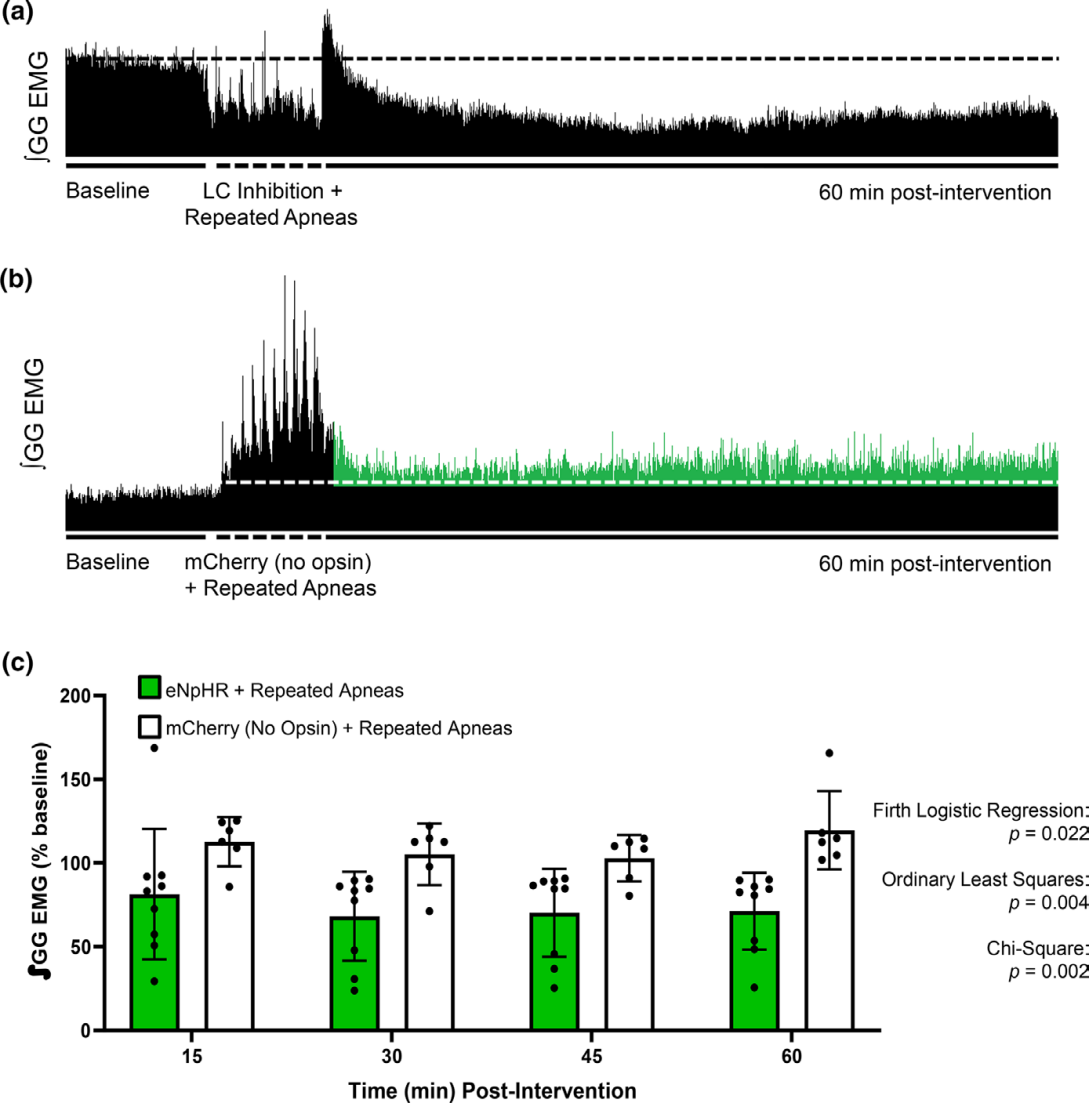
Optical inactivation of the LC prevents apnea‐induced LTF. (a) Integrated inspiratory genioglossus motor output (∫GG EMG) recorded from an anesthetized spontaneously breathing rat, depicting baseline genioglossus amplitude, and the decrease in EMG amplitude during LC inhibition plus the reflexive increase in EMG amplitude during repeated apneas. The subsequent lack of LTF afterwards can also be observed, with the dotted line indicating baseline activity. (b) Integrated inspiratory genioglossus motor output (∫GG EMG) recorded from an anesthetized spontaneously breathing rat, depicting baseline genioglossus amplitude, the increase in EMG amplitude during repeated apneas paired with LC illiumination without opsins, and the subsequent expression of GG LTF. (c) Group data showing inspiratory genioglossus EMG (∫GG EMG) activity in animals that did not express the inhibitory opsin (mCherry, *n* = 6, white bars) increased by 120 ± 8% following repeated apneas (i.e., LTF) (1‐way RM ANOVA, *F* = 2.771, *p* = 0.0466). However, when the LC was inactivated (eNpHR, *n* = 9, green bars), repeated apneas produced a decrease in GG amplitude over time (1‐way RM ANOVA, *F* = 4.874, *p* = 0.0041). When the groups are compared, we found LC inactivation significantly correlated with an absence of hLTF expression (Firth logistic regression, LC inactivation + Apneas vs. mCherry + Apneas, *p* = 0.022; OLS, LC inactivation + apneas vs. mCherry + apneas, *R* ^2^ = 0.3629, F_(17)_=10.82, *β* = −0.40125, *p* = 0.004; chi‐square test, LC Inactivation + Apneas vs. mCherry + Apneas, χ^2^ = 9.6923, *p* = 0.002). Data presented as mean + SD.

Pharmacological inactivation of the LC with clonidine prevented apnea‐induced hLTF (Lui et al., 2018), but an optical approach provides precision and temporal control that is otherwise missing from a pharmacological approach. Repeated apneas can trigger hLTF, thus we aimed to optically inhibit eNpHR‐expressing LC cells during this trigger and found optically inactivated eNpHR‐expressing LC neurons prevented apnea‐induced hLTF (Figure 5). In fact, a significant decrease in genioglossus motor output at 60 min was observed (1‐way RM ANOVA, *F* = 4.874, *p* = 0.0041, Figure 5c) but diaphragm amplitude, respiratory rate, blood oxygen saturation and end‐tidal CO_2_ levels, were unaffected (Figure S4). To ensure that light itself did not inhibit apnea‐induced hLTF expression, the same intervention was used in animals absent eNpHR and repeated apneas were able to elicit hLTF with genioglossus amplitude reaching 20 ± 8% above baseline (1‐way RM ANOVA, *F* = 2.771, *p* = 0.0466, Figure 5b,c). These findings support previous findings that suggest LC activity to be critical for apnea‐induced hLTF (Lui et al., 2018).

Using a Firth logistic regression analysis to model hLTF expression, we found repeated apneas were significantly more likely to induce hLTF expression compared to controls. This suggests that hLTF expression following recurrent apneas was not due to random chance. To statistically verify that LC inactivation prevented apnea‐induced hLTF (i.e., we did not simply have successive nonresponders in our sample set), we again performed a chi‐square test, Firth logistic regression, and an OLS linear regression to model the probability of hLTF expression. To do this, we included all animals that received LC inactivation with repeated apneas, and all animals that received the control virus plus repeated apneas. By including all animals regardless of whether they expressed hLTF following our intervention, we found LC inactivation significantly correlated with an absence of hLTF expression (chi‐square test, LC Inactivation + Apneas vs. mCherry + Apneas, χ^2^ = 9.6923, *p* = 0.002). Similarly, a significant difference was observed in the probability of hLTF expression when the LC is inactivated compared to control (Firth logistic regression, LC inactivation + Apneas vs. mCherry + Apneas, *p* = 0.022). Lastly, upon performing OLS analysis, LC inactivation statistically prevented apnea‐induced hLTF (OLS, LC inactivation + apneas vs. mCherry + apneas, *R* ^2^ = 0.3629, F_(17)_=10.82, *β* = −0.40125, *p* = 0.004). Taken together, this suggests that the LC is critical for apnea‐induced hLTF.

### 3.8 Noradrenaline release following LC stimulation is the critical neurotransmitter mechanism mediating hypoglossal LTF

**FIGURE 6:**
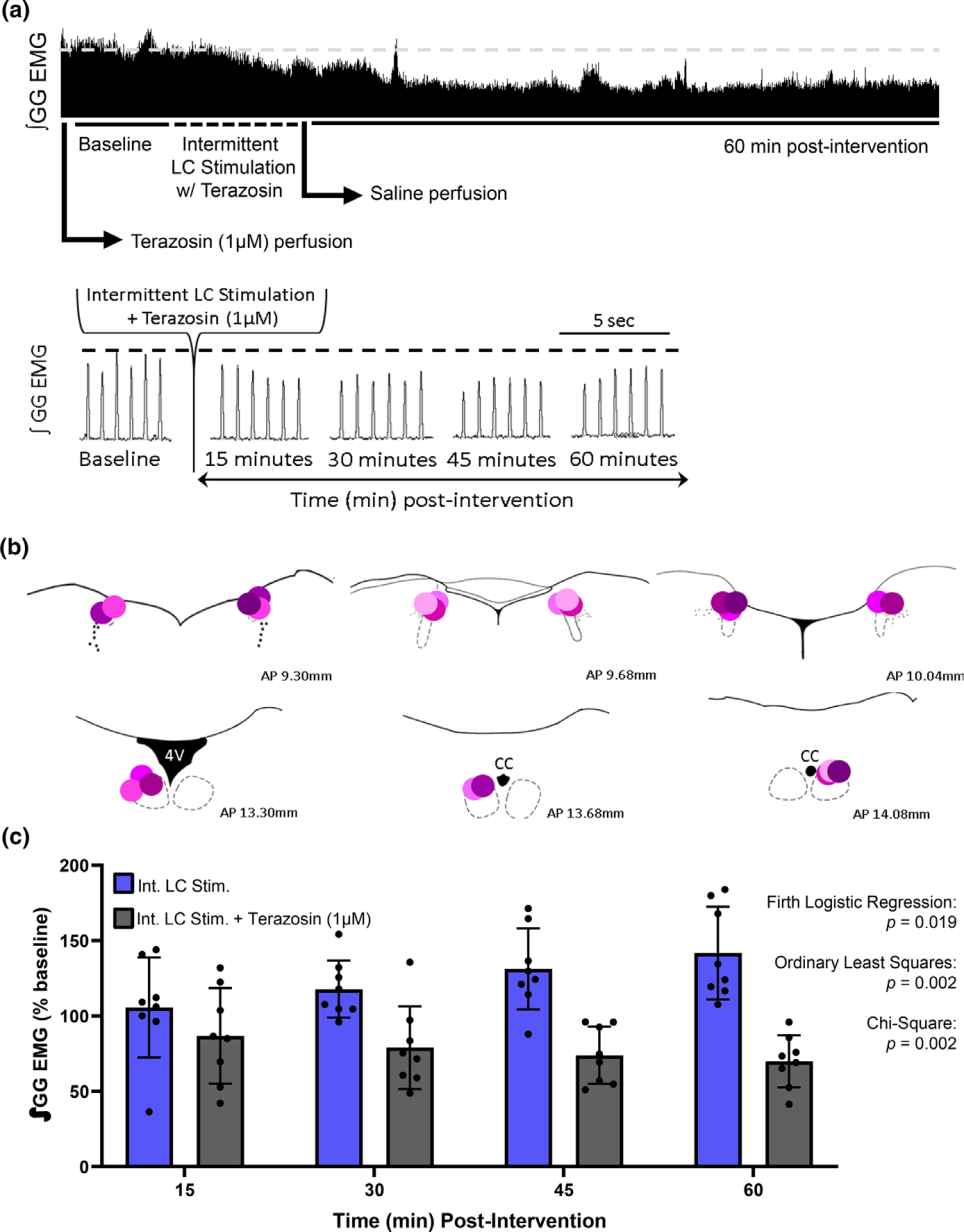
LTF is mediated by α1‐adrenergic receptor binding of noradrenaline released from the LC. (a) Representative EMG trace of integrated inspiratory genioglossus (GG) activity after α1‐adrenergic receptor blockade at the level of the hypoglossal motor pool, followed by intermittent LC stimulation. Terazosin perfusion was replaced by saline for the remainder at the recording over 60 min. Below is the magnified EMG trace shown at baseline after terazosin perfusion, and a magnified example of GG activity at 15, 30, 45, and 60 min time‐points. (b) Probe tract locations in the LC (dotted outline, left panels) and in the hypoglossal motor pool (dotted outline, right panels) following LC stimulation and terazosin perfusion. Matching shade of pink/purple represents probe locations in the LC and hypoglossal motor pool within each animal. (c) Group data showing inspiratory genioglossus activity of animals perfused with the α1‐adrenergic blocker (*n* = 8, black bars) could not exhibit LTF following intermittent LC stimulation, but instead induced a decrease in genioglossus amplitude (1‐way RM ANOVA, *F* = 3.737, *p* = 0.0166). When the groups are compared, a significance difference in the probability of hLTF expression is observed (Firth logistic regression, Int. LC Stim. vs. Int. LC Stim. + Teraz, *p* = 0.019. OLS, Int. LC Stim. vs. Int. LC Stim. + Teraz, *R* ^2^ = 0.3272, F_(30)_=7.782, *β* = 0.9541, *p* = 0.002; chi‐square test, Int. LC Stim. vs. Int. LC Stim. + Teraz, χ^2^ = 10.05, *p* = 0.002). Data presented as mean + SD.

Next, repeated optical stimulation of ChR2‐expressing LC cells was performed while perfusing 1 μM terazosin, an α1‐adrenergic receptor antagonist, at the hypoglossal motor pool (Figure 6). When α1‐noradrenergic receptors on the hypoglossal motor pool were blocked, intermittent LC stimulation did not trigger hLTF. In fact, a significant decrease in genioglossus motor output was observed (1‐way RM ANOVA, *F* = 3.737, *p* = 0.0166, Figure 6c). No other respiratory variables measured differed from baseline in all groups (Figure S5). The absence of hLTF, therefore, suggests that the release of noradrenaline from the LC must act on α1‐noradrenergic receptors at the hypoglossal motor pool for hLTF to manifest.

Using a chi‐square test, a Firth logistic regression, and an OLS, we again tested to determine whether our intervention truly had an impact on influencing the probability of hLTF expression. We found that intermittent LC stimulation elicited hLTF 73% of the time. When we perfused terazosin, we found that terazosin perfusion did successfully prevent hLTF, and this result was significantly correlated with the LC stimulation intervention (chi‐square test, Int. LC Stim. vs. Int. LC Stim. + Teraz, χ^2^ = 10.05, *p* = 0.002. Firth logistic regression, Int. LC Stim. vs. Int. LC Stim. + Teraz, *p* = 0.019. OLS, Int. LC Stim. vs. Int. LC Stim. + Teraz, *R* ^2^ = 0.3272, *F*_(30)_=7.782, *β* = 0.9541, *p* = 0.002). Taken together, all three statistical analyses support the same finding, suggesting that the LC‐induced hLTF is mediated through noradrenergic mechanism with noradrenaline release from the LC onto α1‐noradrenergic receptors on the hypoglossal motoneurons.

### 3.9 Apnea‐induced hLTF requires persistent α1‐noradrenergic receptor activation after induction

**FIGURE 7:**
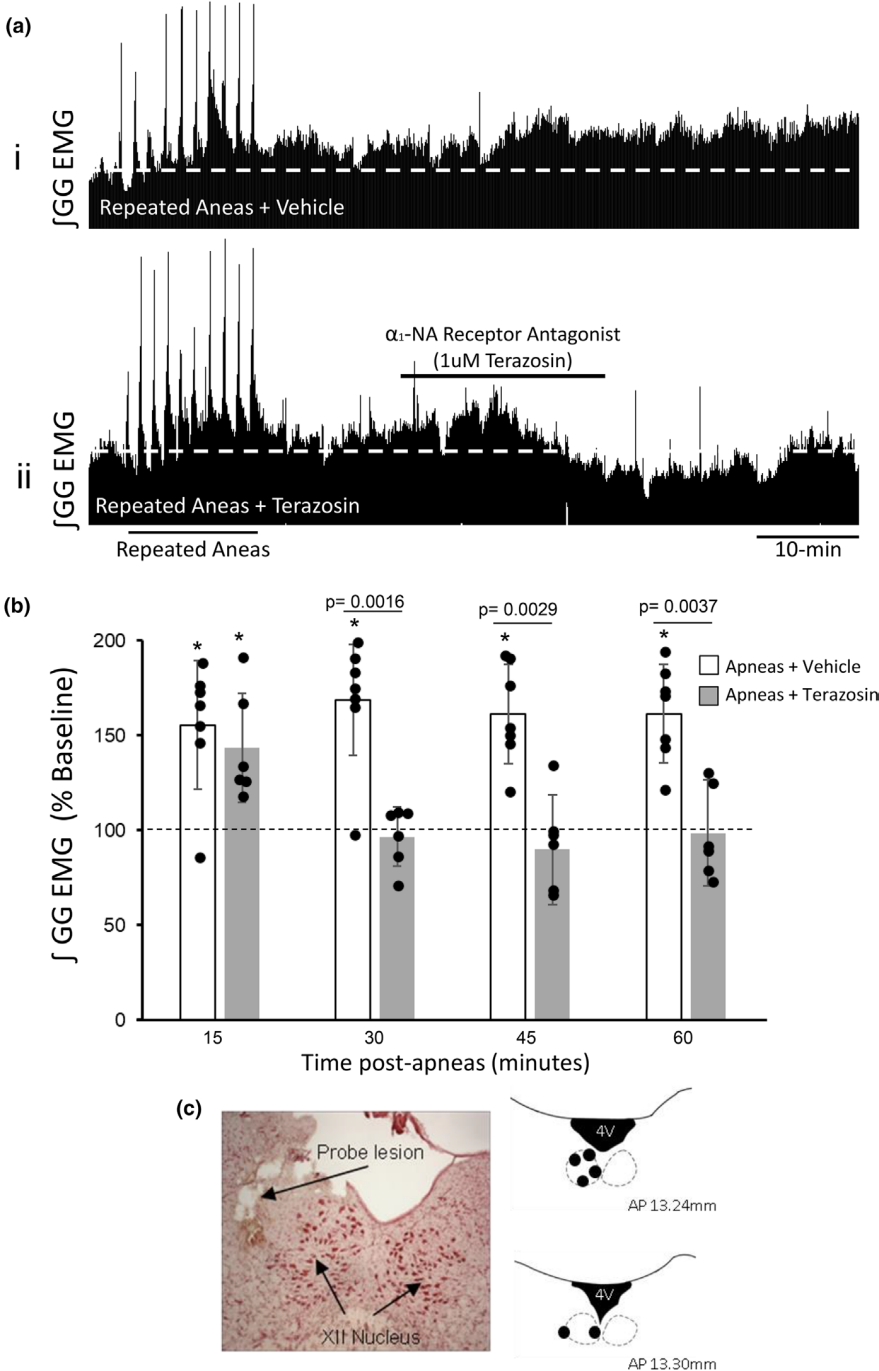
α1‐ Noradrenergic receptor activation is required for maintenance of apnea‐induced hypoglossal long‐term facilitation (LTF). (a) Representative integrated inspiratory genioglossus EMG recordings before, during and for 60 min after repeated apneas. Top trace (i) demonstrates LTF of hypoglossal motor output in response to repeated apneas in a vehicle treated rat. Bottom trace (ii) demonstrates that apnea‐induced LTF is abolished when α1‐noradrenergic receptors are blocked at the hypoglossal motor pool *after* repeated apneas. (b) Mean values expressed as a percentage change from baseline (baseline = 100), showing that repeated apneas trigger LTF of hypoglossal motor output; at 60‐min post repeated apneas, inspiratory genioglossus EMG activity was significantly elevated above baseline levels (white bars). In separate group of rats, perfusion of 1 μM terazosin at the hypoglossal motor pool *following* repeated apneas abolishes the expression of hypoglossal LTF (gray bars), demonstrating that apnea‐induced hypoglossal LTF requires α1‐ noradrenergic receptor activation for its *maintenance*. (c) Microdialysis probes are located in the hypoglossal motor pool. Black filled circles represent the tip of the probe lesions in the hypoglossal motor pool. Group data are expressed as a percentage change from baseline ± SD. Asterisks (*) denote a significant difference (*p* < 0.05) compared to baseline at the indicated timepoint within each respective group. Indicated *p* values denotes a significant difference between the two experimental groups at the indicated time points.

While hLTF is mediated by noradrenaline release onto hypoglossal motoneurons following LC stimulation, it remains unknown whether noradrenaline is only needed to initiate hLTF, and/or whether it is also needed to sustain/maintain hLTF (i.e., initiation versus maintenance). Here, we addressed this question by blocking α1‐noradrenergic receptors at the XII, *after* the repeated apnea protocol. First, we show that repeated apneas could trigger hLTF in animals that received vehicle/saline perfusion at the hypoglossal motor pool (61 ± 26% above baseline levels at 60‐min post‐apneas, *p* = 0.0008, 1‐way RM ANOVA Figure 7a,b). In a separate group of animals, blocking noradrenergic receptors at the hypoglossal motor pool after repeated apneas prevented the maintenance of hypoglossal LTF (Figure 7a,b). At 15 min following repeated apneas, genioglossus muscle tone was elevated by 44 ± 28% above baseline levels (1‐way RM ANOVA, *p* < 0.05; *n* = 6). Perfusion of 1 μM terazosin at the 15‐min timepoint after apneas had a significant effect on the development of apnea‐induced hLTF. Specifically, elevated inspiratory genioglossus muscle activity following repeated apneas was returned to baseline and remained at baseline during the remainder of the experimental recording period (1.5 ± 11% below baseline at 60‐min post‐apneas, *p* > 0.05). This finding demonstrates that persistent noradrenergic receptor activation at the hypoglossal motor pool is necessary for maintenance of apnea‐induced hLTF. Like previous findings, diaphragm amplitude, respiratory rate, blood oxygen saturation, and end‐tidal CO_2_ levels were unaffected. Lastly, postmortem histology was used to confirm placement of probes in the hypoglossal motor pool (Figure 7c).

In a separate group of time‐matched control rats that did not get exposed to repeated apneas, we show that perfusion of terazosin alone at the hypoglossal motor pool did not influence basal inspiratory genioglossus muscle activity at all time points tested (12 ± 23% below baseline levels at 60 min after 1 μM terazosin perfusion, *p* = 0.255). This suggests that terazosin‐induced reversal of hLTF following repeated apneas at the given dose tested was not due to mere dis‐facilitation of excitatory hypoglossal motor activity per se.

### 3.10 Intact Trk receptor signaling is necessary for the expression of apnea‐induced upper airway motor plasticity

α1‐noradrenergic receptor activation involves signaling through Gq‐protein coupled receptors that activate downstream pathways regulated by phospholipase C, IP3 kinase, and protein kinase C, which in turn function to phosphorylate/activate other proteins (Perez, 2020; Rosenbaum et al., 2009). In addition, this pathway is known to be coupled to the activation of neurotrophin pathways, including BDNF, that signal through tropomyosin‐related kinase (Trk)‐B receptors (Chen et al., 2007; Counts & Mufson, 2010; Dale et al., 2013; Huang & Reichardt, 2003; Leal et al., 2014; Lee & Chao, 2001). These combined events can produce excitatory cellular effects that can manifest as an increase in synaptic strength. Here, we aimed to determine whether apnea‐induced, noradrenergic‐dependent hypoglossal LTF requires activation of Trk receptors at the level of hypoglossal motor pool. Therefore, we focally blocked Trk receptors via application of K252a (Trk receptor inhibitor) at the hypoglossal motor pool 20 min prior to our repeated apnea protocol. Following this intervention, we found that pretreatment with K252a at the hypoglossal motor pool (10 μM or 50 μM; *n* = 6 and *n* = 7, respectively) prevented apnea‐induced LTF of genioglossus muscle tone (Figure S6; 3.2 ± 18% and 12 ± 19% below baseline levels at 60 min for 10 μM and 50 μM k252a, respectively; 1‐way RM ANOVA, *p >* 0.05 at all time‐points tested). No long‐term changes in diaphragm EMG activity, respiratory rate, blood oxygen saturation, or end‐tidal CO_2_ were observed. Taken together, these findings demonstrate that in addition to requiring noradrenergic receptor activation before and after repeated apneas, apnea‐induced hLTF also requires activation of Trk receptors at the hypoglossal motor pool.

### 3.11 Adenosine 2a receptor activation at the hypoglossal motor pool is sufficient for triggering hLTF and requires noradrenergic signaling

**FIGURE 8:**
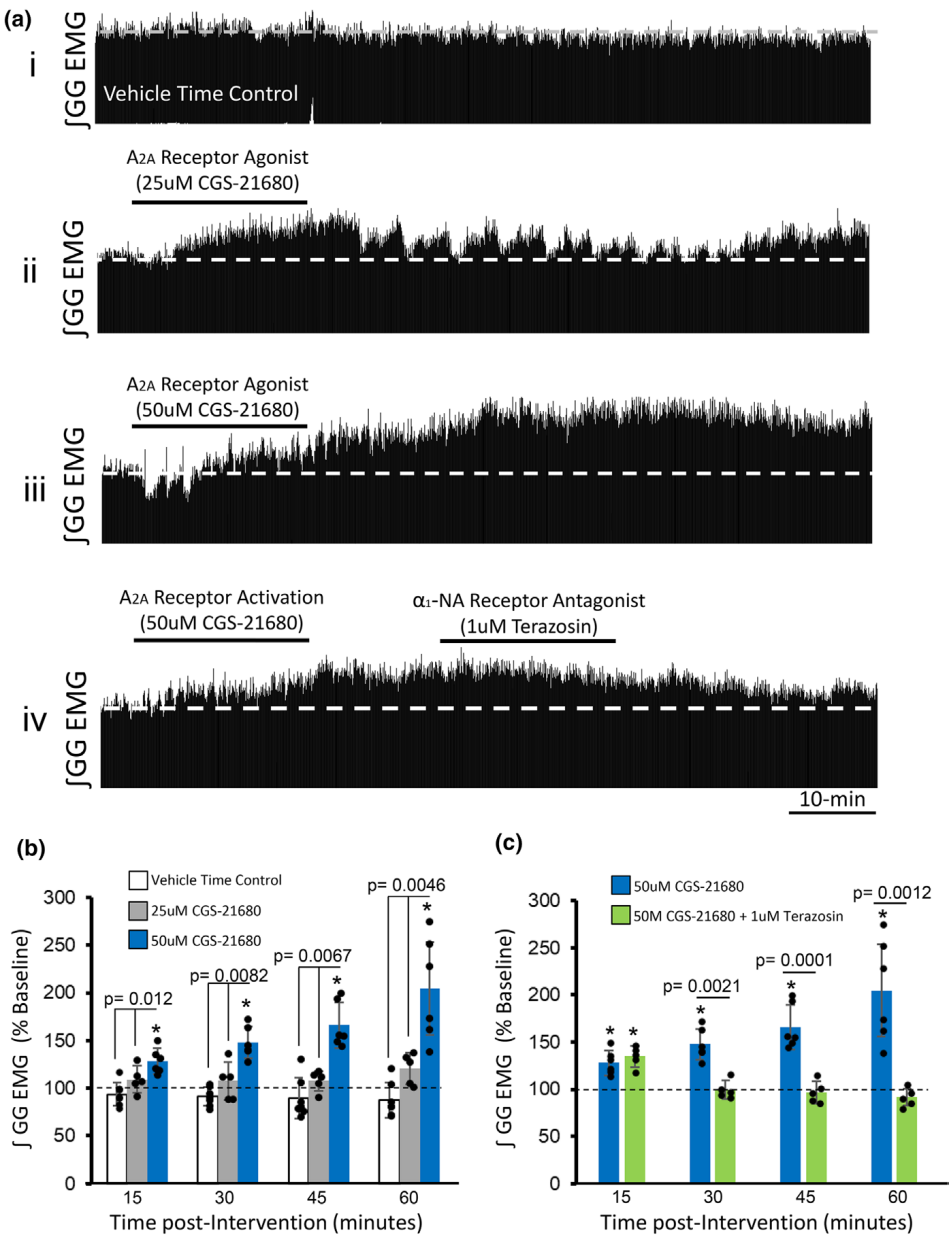
Adenosine‐2a receptor activation at the hypoglossal motor pool triggers hypoglossal long‐term facilitation (LTF) and requires α1‐noradrenergic‐dependent processes. (a) Representative integrated inspiratory genioglossus (GG) EMG recordings in a time‐matched vehicle treated rat (i), and in rat subjects that received either 25 μM (ii) or 50 μM (iii) CGS‐21680 perfusion (adenosine 2A receptor agonist) at the hypoglossal motor pool. Bottom trace (iv) demonstrates integrated inspiratory GG EMG activity in a rat subjected to 50 μM CGS‐21680 followed by 1 μM terazosin perfusion at the hypoglossal motor pool. While 50 μM CGS‐21680 perfusion alone at the hypoglossal pool triggers robust LTF of hypoglossal motor output (iii), antagonism of α1‐noradrenergic receptors (via terazosin) at the hypoglossal motor pool *following* CGS‐21680 perfusion abolishes the expression of hypoglossal LTF (iv). (B) Mean values expressed as a percentage change from baseline (baseline = 100), showing that activation of adenosine 2a receptors via perfusion of 50 μM CGS‐21680 at the hypoglossal motor pool triggers LTF of inspiratory GG motor activity (blue bars): At 60‐min post perfusion, inspiratory GG EMG activity was significantly elevated compared to baseline levels (*p* < 0.05). (c) In a separate group of rats that received 1 μM terazosin perfusion following 50 μM CGS‐21680 perfusion at the hypoglossal motor pool, hypoglossal LTF failed to develop (green bars): Inspiratory GG EMG activity remained at baseline levels at 60‐min post perfusion, demonstrating that adenosine‐2a receptor‐induced hypoglossal LTF requires α1‐ noradrenergic receptor activation for its maintenances. Group data are expressed as a percentage change from baseline ± SD (baseline = 100). Asterisks (*) denote a significant difference (*p* < 0.05) compared to baseline at the indicated timepoint within each respective group. Indicated *p* values denote a significant difference between rats that received 50 μM CGS‐21680 perfusion alone and the other experimental groups at the indicated time points.

Since apnea‐induced hLTF requires Trk receptor activation at the hypoglossal motor pool, we aimed to determine whether activation of Trk receptors within hypoglossal motoneurons alone is sufficient at eliciting hLTF, and if so, does it require noradrenergic signaling. An adenosine A2a receptor agonist (CGS21680), known to trans‐activate Trk receptors (Lee & Chao, 2001; Sebastião & Ribeiro, 2009; Wiese et al., 2007) was used as a pharmacological tool to activate Trk signaling at the hypoglossal motor pool. Following focal perfusion of 50 μM CGS21680 within the hypoglossal motor pool, we observed a sustained enhancement in integrated inspiratory genioglossus muscle tone, lasting for at least 1 h, increasing by 104 ± 48% above baseline levels at 60‐min post perfusion (1‐way RM ANOVA; *p* < 0.05. Figure 8a,b). Drug effects had no long‐term changes in diaphragm EMG activity, respiratory rate blood oxygen saturation, or end‐tidal CO_2_ levels. To control for the effect of microdialysis perfusion alone, a separate group of time‐matched control rats were subjected to only saline perfusion at the hypoglossal motor pool. In this group, integrated inspiratory genioglossus activity remained close to baseline levels through the recording period (12 ± 18% below baseline at 60‐min post‐perfusion, *p* > 0.05).

To determine whether adenosine 2a‐receptor‐induced hLTF requires noradrenergic signaling, we antagonized α‐1 noradrenergic receptors at the hypoglossal motor pool following adenosine‐A2a‐receptor activation. We found that adenosine‐A2a‐receptor‐induced hypoglossal motor facilitation was rapidly reversed following perfusion with terazosin—an α1 noradrenergic receptor antagonist (Figure 8c). At 15 min following CGS21680 perfusion, integrated inspiratory genioglossus activity was significantly increased by 34 ± 11% above baseline levels (1‐way RM ANOVA, *p* < 0.05). However, this enhancement was rapidly reversed following terazosin perfusion at the hypoglossal motor pool; genioglossus inspiratory amplitude returned to baseline levels and remained unchanged over the remainder of the experimental recording period (9 ± 10% below baseline at 60 min; 1‐way RM ANOVA, *p* > 0.05). Taken together, these findings demonstrate that (1) activation of Trk receptor signaling is sufficient for eliciting hypoglossal motor facilitation, and (2) Trk receptor signaling and noradrenergic‐mediated processes work in a cooperative manner in the regulation of hypoglossal motor plasticity.

## 4 DISCUSSION

Long‐term facilitation of inspiratory genioglossus motor output can be elicited by various triggers such as repeated bouts of hypoxia, repeated obstructive apneas, or inactivity‐induced motor facilitation (Baertsch & Baker‐Herman, 2013; Tadjalli et al., 2010; Wilkerson et al., 2018). Here we presented two novel triggers that can elicit hLTF without direct exposures to respiratory challenges within the respiratory system. Intermittent stimulation of ChR2‐expressing LC neurons or pharmacological activation of Trk signaling at the hypoglossal motoneurons both triggered robust hLTF (Figures 3,8). Furthermore, we provide evidence to suggest that the LC mediates hLTF through a noradrenergic‐dependent mechanism, and persistent noradrenergic stimulation is necessary for maintenance of hLTF expression. Together, our findings identify the LC as the key neuroanatomical source for the relevant noradrenaline necessary for the expression of hLTF and provide new insights into the cell signaling mechanisms that underlie plasticity of upper airway respiratory motor activity.

### 4.1 Stimulation of ChR2‐expressing LC neurons trigger LTF of inspiratory genioglossus motor output

Here, we show that intermittent LC stimulation triggers hLTF. However, repeated application of noradrenaline on hypoglossal motoneurons in vitro potentiates their response to excitatory inputs (Neverova et al., 2007). It may be possible that our stimulation protocol forced intermittent release of noradrenaline from the LC onto the hypoglossal motor pool, thereby triggering hLTF. This intervention could, therefore, simply be an observation of an already known phenomenon (i.e., that repeated exposure to noradrenaline can trigger hLTF). However, repeated exposure of noradrenaline from non‐LC sources could not elicit sustainable (i.e., >60 min) hLTF (Song & Poon, 2017) Here, we show that targeting LC neurons triggers a form of hLTF that persists for a minimum of 60 min, suggesting that repeated stimulation of the LC elicit hLTF that is reflective of the plasticity observed with the repeated apnea trigger.

### 4.2 LC provides an endogenous noradrenergic drive to hypoglossal motoneurons

Genioglossus motor output is under noradrenergic state‐dependent modulation (Chan et al., 2006), and one source of noradrenaline is the LC as it has direct projections to the hypoglossal motor pool (Lui et al., 2018). Here, we provide evidence demonstrating the presence of an endogenous noradrenergic drive arising from the LC onto hypoglossal motoneurons. By using an optogenetic approach, inactivation of LC cells was immediately accompanied by a decrease in basal inspiratory genioglossus muscle EMG amplitude. Despite reports of limited innervations between LC and XII motoneurons (Aldes et al., 1992; Rukhadze & Kubin, 2007), these findings suggest that a portion of the tonic noradrenergic drive at the hypoglossal motor pool stems from the LC. In contrast, genioglossus motor output increases following pharmacological application of phenylephrine or norepinephrine (Chan & Sawchenko, 1994; Neverova et al., 2007; Parkis et al., 1995), but direct LC stimulation did not increase genioglossus activity. It may be possible that direct LC optogenetic stimulation may not mimic the aforementioned pharmacological studies, and hence, it may not induce sufficient release of noradrenaline that immediately augments hypoglossal motoneuronal excitability. Similarly, no change in inspiratory genioglossus motor output was observed in a study that optically stimulated other noradrenergic groups (A5 and A7), which reportedly has greater noradrenergic innervations to the hypoglossal motor pool than the LC (Song & Poon, 2017).

### 4.3 A minimum threshold of LC stimulation is required for hLTF expression

In some animals, we reported that our intervention (repeated apneas or intermittent LC stimulation) did not trigger hLTF. Our investigation into why the response in these animals differed led us to changes observed in histology. Despite receiving the same intervention, nonresponders did not receive the same level of stimulation, suggested by a difference in as c‐Fos expression observed in the LC. LC neurons have been suggested to fire synchronously (Aston‐Jones & Bloom, 1981; Christie et al., 1989; Ishimatsu & Williams, 1996), and it may, therefore, be possible that an insufficient number of cells were stimulated to trigger synchronous firing and thereby hLTF. The difference in stimulation may be a result of variation in our hands, or simply different responses to a fixed stimulus (i.e., consistently 10 periods of LC stimulation at 5 Hz for 15 s, separated by 1 min), as our protocol does not account for the variability between each animal. Taken together, it suggests that a minimum threshold of LC stimulation must be met to trigger synchronous firing and therefore hLTF expression.

### 4.4 Noradrenergic‐dependent processes are involved in mediating hLTF expression across multiple triggers

The idea that upper airway respiratory motor plasticity requires noradrenergic‐dependent processes has been the subject of investigation in the past (Feldman et al., 2005; Lui et al., 2018; Neverova et al., 2007; Tadjalli et al., 2010). Here, we show that α1‐noradrenergic receptors are required for hLTF expression regardless of the modality of the stimulus used. This is supported by our findings that show α1‐noradrenergic receptor activation is required for initiation, as well as maintenance of hLTF. Specifically, apnea‐induced hLTF is abolished when α1‐noradrenergic receptors are blocked during apneas (Tadjalli et al., 2010) or after apneas (Figure 7). In addition, hLTF elicited by either repeated apneas, intermittent LC stimulation, or adenosine‐A2a‐receptor agonism ultimately requires the activation of α1‐noradrenergic receptors at the hypoglossal motor pool. Another study demonstrated that hLTF elicited by repeated episodes of hypoxia was also abolished upon systemic α1‐noradrenergic receptor blockade (Neverova et al., 2007). Taken together, this suggests that noradrenaline, and specifically α1‐noradrenergic receptor activation, is critical for hLTF regardless of the trigger (i.e., repeated apneas, repeated episodes of hypoxia, repeated LC stimulation, or activation of Trk receptor signaling via adenosine 2a receptors at the hypoglossal motor pool).

### 4.5 Comparisons of apnea‐induced hypoglossal LTF and hypoxia‐induced phrenic LTF

Both hypoglossal and phrenic LTF can be induced by various triggers (Bocchiaro & Feldman, 2004; Lui et al., 2018; Mateika et al., 2018; Neverova et al., 2007; Tadjalli & Mitchell, 2019) but the mechanisms underlying these triggers can vary. For example, phrenic LTF (pLTF), a form of serotonin‐dependent plasticity, is induced by intermittent bouts of hypoxia (Mateika et al., 2018; Ostrowski et al., 2023; Tadjalli & Mitchell, 2019; Wilkerson & Mitchell, 2009). Intermittent hypoxia has also been shown to induce hLTF (McKay et al., 2004; Neverova et al., 2007). However, repeated apneas, which evoke a weaker and shorter bout of hypoxia, only trigger hLTF suggesting that intermittent hypoxia and repeated apneas may engage different neural pathways/mechanisms for plasticity induction. Our results show that the neurochemical mechanism and structures involved in hLTF expression are unique to the upper airways and do not involve plasticity induction at the level of phrenic motor pool. Specifically, the neuromodulator and key critical structures for hLTF elicited by repeated apneas are noradrenaline and the LC, whereas it has previously been shown that pLTF induced by intermittent hypoxia involves serotonin and caudal raphe neurons (Bach & Mitchell, 1996; Dodig et al., 2012; Valic et al., 2010).

Due to the lack of connections observed between the LC and the phrenic motor pool, it is not surprising that direct LC stimulation has no long‐term effects on phrenic motor output. Even in the context of intermittent hypoxia, it has been shown that systemic antagonism of adrenergic receptors with prazosin abolishes the expression of hLTF but not LTF of phrenic motor output (Huxtable et al., 2014). Although the referenced study performed by Huxtable et al. (2014) did not identify the key neuroanatomical sites for the actions of noradrenaline (systemic prazosin could have acted at multiple sites throughout the neuroaxis), they provided some limited evidence that differing cellular mechanisms underlying LTF may operate in different motor neuron pools. Indeed, different cellular mechanisms may underlie LTF expression across different respiratory motor pools since our earlier study by Tadjalli et al. (2010) showed that noradrenergic, but not serotonergic receptor activation was necessary for initiation of apnea‐induced hLTF at the hypoglossal motor pool. More importantly, we provide further evidence that noradrenergic receptor activation is also required for maintenance of apnea‐induced hLTF (Figure 7). This profoundly differs from pLTF, which requires serotonin receptor activation for its initiation, but not maintenance (Fuller et al., 2001). This finding, together with our previous published work, suggests that the dominant endogenous neurotransmitter systems involved in the expression of hypoglossal LTF may differ from those that orchestrate phrenic LTF (e.g. serotonin). While apnea‐induced hLTF and intermittent hypoxia‐induced phrenic LTF differ in their requirement for the type of neurotransmitter (i.e., noradrenaline versus serotonin), they do share some similarities. For example, both forms of respiratory motor plasticity require intermittent, but not continuous stimuli. In addition, both forms of motor plasticity also require Trk receptor signaling (Dale et al., 2017).

### 4.6 Pathophysiological significance

Upper airway resistance, compliance, and collapsibility are important determinants of pulmonary function, and appropriate neuromuscular control of lingual motor tone is necessary for maintenance of pharyngeal muscle patency and ventilation (Gold & Schwartz, 1996; Hudgel et al., 1987; Patil et al., 2007; Sankri‐Tarbichi et al., 2009; Wiegand et al., 1989). More importantly, adequate patency of the upper airways has been identified as one of the key therapeutic targets in a number of clinical conditions, including sleep disordered breathing and spinal cord injury (Horner & Peever, 2017; Horton et al., 2017; Sankari et al., 2014; Sankri‐Tarbichi et al., 2009; Veasey & White, 2013; White, 2005; Young et al., 2002). It is proposed that identification and characterization of the mechanisms responsible for pharyngeal motor control and upper airway collapse are needed to develop effective therapies that preserve upper airway patency. Restoration, repair, and rehabilitation of upper airway motor output is a priority because of its profound influence on pulmonary ventilation. Therefore, effective neurochemical modulation of pharyngeal muscle tone is dependent on the identification of the relevant neurotransmitters and receptors, and in clinical contexts, it becomes a key translational objective. In our study, we show that noradrenaline release triggered by LC activation initiates downstream signaling cascades necessary to enhance inspiratory activity of the genioglossus muscle (a major pharyngeal dilator). We suggest that manipulation of this circuitry may provide novel treatments for clinical conditions that compromise upper airway motor activity, and therefore warrant future studies to further characterize neural circuits and receptor signaling pathways that regulate upper airway motor activity.

### 4.7 Methodological considerations

As with any approach, there were limitations to our methodology. To start, our use of c‐Fos as a marker for cell activity does not provide insight into cells that have been inactivated or inhibited (Chan & Sawchenko, 1994). Furthermore, not all activated neurons express c‐Fos. As a result, our claims on LC activity following LC stimulation or inhibition may not truly reflect the real‐time activities of the cell before, during, or after hLTF induction. It can only provide a snapshot of what may be occurring at the LC during my intervention. In addition, noradrenaline levels were not measured in these studies following stimulation or inhibition of LC cells.

Another consideration includes our use of 1 μM terazosin perfused into the hypoglossal motor pool via reverse microdialysis. Although direct perfusion of the drug via reverse microdialysis is far more precise and superior to an intrathecal approach (used by other research groups), it is possible that terazosin perfusion affected α1‐noradrenergic receptors found on NTS neurons (Zhang & Mifflin, 2007), and that this interaction influenced the expression of hLTF. However, α1‐noradrenergic receptors on nucleus tractus solitarius neurons are not tonically active (Zhang & Mifflin, 2007) and its antagonism should therefore have no influence on hLTF expression. Lastly, we did not investigate how LC manipulation affected blood pressure or heart rate. LC stimulation could potentially decrease blood pressure and heart rate (Hakuno et al., 2004; Sved & Felsten, 1987). It may therefore be possible that repeated modulation of these factors contributed to the manifestation of hLTF. However, we have previously measured mean arterial blood pressure between groups and found no observable difference between animals that developed hLTF and those that did not (Lui et al., 2018).

## 5 CONCLUSION

We provide evidence that the LC is a key neuroanatomical structure involved in the circuitry that triggers hLTF following repeated apneas. The involvement of the LC is an important discovery because forced noradrenaline release from other noradrenergic cell groups such as the A5 and A7 only augments inspiratory genioglossus motor output for 20 min (Song & Poon, 2017). It is possible that persistent release of noradrenaline from the LC, specifically, is required to sustain hLTF for longer durations, implying that plasticity at the LC is necessary for persistent hLTF. Our hypothesized neural circuit mediating hLTF initially detailed in Lui et al. (2018) suggested that the LC could be a key structure. Repeated obstructive apneas would elicit chemical and mechanical feedback. This signal is carried by the vagus nerve and terminates largely in the nucleus tractus solitarius, which in turn innervates noradrenergic LC neurons to act on the hypoglossal motor pool by releasing noradrenaline on hypoglossal motoneurons. This in turn initiates a set of cellular cascades leading to manifestation of hLTF. Here, we confirm that the LC is a key player in this neural circuit, and that the LC's continual contribution during hLTF is essential for hypoglossal motor facilitation. Lastly, we also provide evidence that noradrenergic receptor activation is not only required for initiation of hLTF, but it is also necessary for its maintenance. Downstream signaling cascades following noradrenergic receptors involve Trk receptor signaling and more importantly, activation of Trk signaling via adenosine 2a receptors is sufficient at eliciting hypoglossal motor facilitation.

## AUTHOR CONTRIBUTIONS

SL, AT, and JP performed the conception and the design of the research. SL wrote the first draft of the manuscript and generated the data shown in Figures 1, 2, 3, 4, 5, 6. AT generated the data and the analysis of the data shown in Figures 7,8. JF assisted with the statistical analyses. SL, AT, and JP reviewed and edited the manuscript.

## CONFLICT OF INTEREST STATEMENT

The authors declare no competing financial interests.

## ETHICS STATEMENT

All procedures and experimental protocols were approved by the Institutional Animal Care and Use Committee at the University of Toronto.All experiments performed in this manuscript also conform to the principles and regulations as described by the Canadian Council on Animal Care. Additionally, all studies are reported in accordance with ARRIVE guidelines. All anesthetized animals were sacrificed via anesthetic isoflurance overdose and transcardial perfusion, permanently terminating heart rate, blood pressure and respiratory activity.

## Supporting information


Figures S1–S6.

## Data Availability

Source data for this study are openly available at DOI: https://doi.org/10.21203/rs.3.rs‐2758520/v1.
