# Supplementary material for: Identification of multiple hypoxia‐independent triggers of upper airway long‐term facilitation in a rat model of upper airway motor plasticity
Source: Physiol Rep. 2025 Mar 11;13(5):e70142. doi: 10.14814/phy2.70142 (PMC11895832; doi:10.14814/phy2.70142)
Supplement: Supplementary file 1 — Figures S1–S6. [file PHY2-13-e70142-s001.pdf]

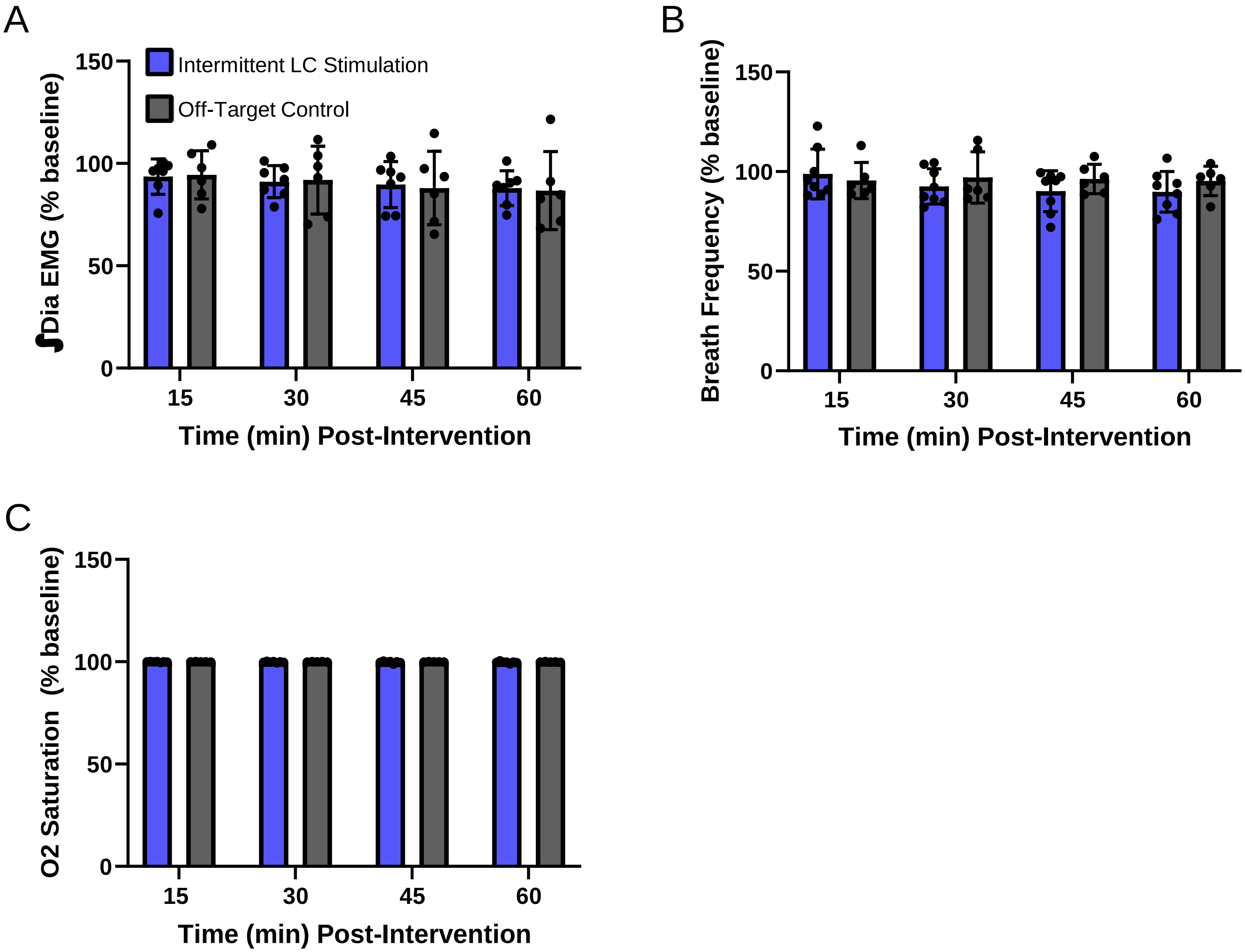
A
Intermittent LC Stimulation
Off-Target Control
∫Dia EMG (% baseline)
150
100
50
0
15
30
45
60
Time (min) Post-Intervention
B
Breath Frequency (% baseline)
150
100
50
0
15
30
45
60
Time (min) Post-Intervention
C
O2 Saturation (% baseline)
150
100
50
0
15
30
45
60
Time (min) Post-Intervention

Supplementary Figure 1 - Intermittent LC Stimulation vs Off-Target LC Stimulation – Diaphragm activity (A), respiratory frequency (B), and oxygen saturation (C) did not differ between rats given ChR2-LC stimulation (blue bars) vs Off-Target stimulation (grey bars) (2-way RM ANOVA, diaphragm: F=0.5053, p=0.7320, respiratory frequency: F=1.329, p=0.2726, O_2 saturation: F=0.5756, p=0.6820). Data presented as mean + SEM.

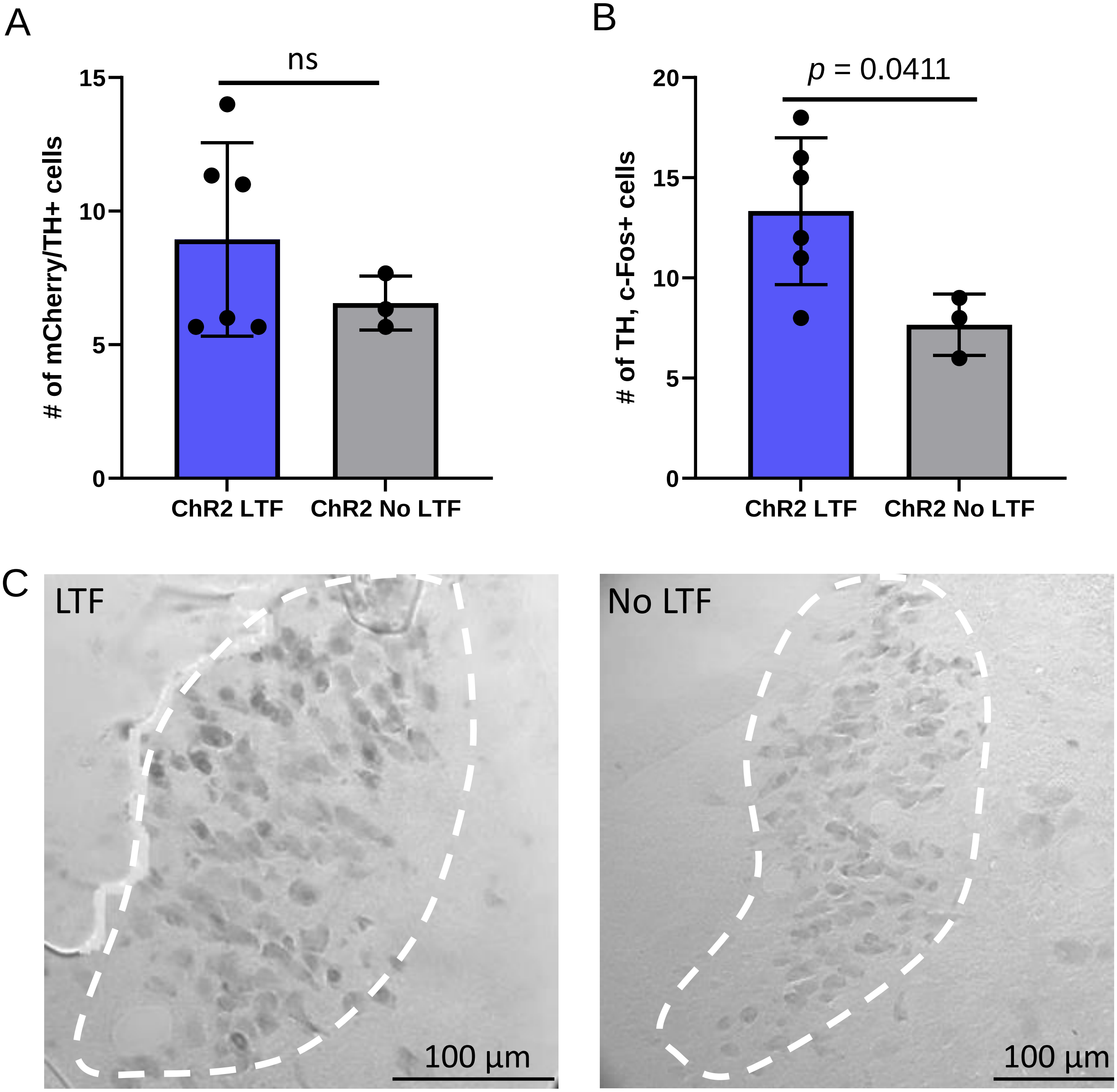
A
ns
# of mCherry/TH+ cells
15
10
5
0
ChR2 LTF
ChR2 No LTF
B
p = 0.0411
# of TH, c-Fos+ cells
20
15
10
5
0
ChR2 LTF
ChR2 No LTF
C
LTF
100 µm
No LTF
100 µm

Supplementary Figure 2. LTF requires a minimum LC stimulation threshold. (A) Group data showing equal amounts of mCherry and tyrosine hydroxylase (TH) expression in animals that expressed LTF (Int. LC Stim. (LTF), n=6, blue bar) compared to animals that did not express LTF (Int. LC Stim. (No LTF), n=3, grey bars) (unpaired t-test, LTF vs No LTF, $t_{(7)}=1.086$, $p=0.3134$). (B) Group data showing animals that exhibited LTF had 43 ± 11% more c-Fos and TH positive cells compared to animals that did not express LTF (unpaired t-test, ChR2 LTF vs ChR2 No LTF, $t_{(7)}=2.499$, $p=0.0411$), suggesting non-responders had insufficient LC stimulation. (C) An example of LC activity represented by c-Fos expression (black nuclei) in animals given intermittent LC stimulation and exhibiting LTF (left) or intermittent LC stimulation but did not exhibit LTF (right), suggesting . Dotted outline identifies the LC. Data presented as mean + SD.

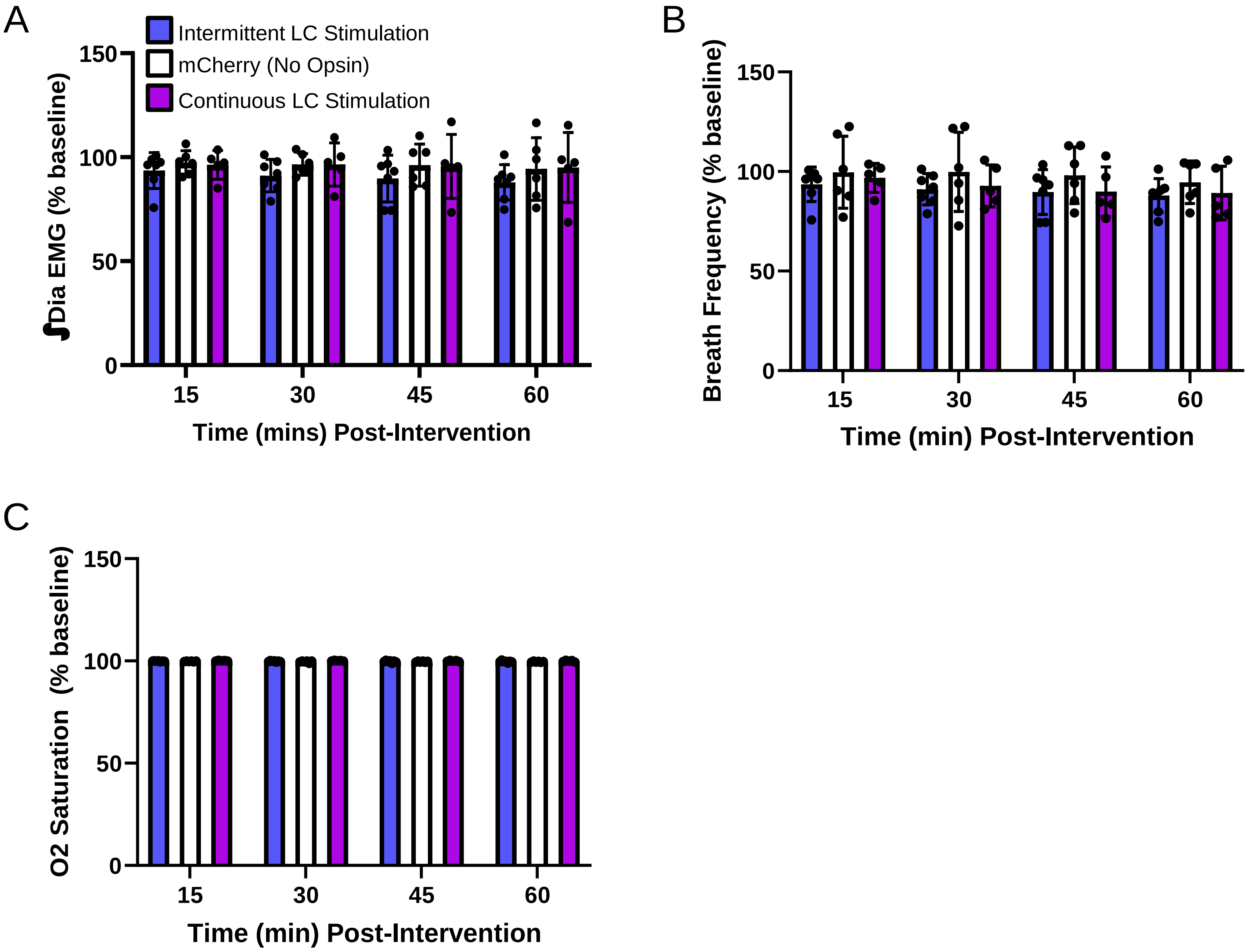

A
Intermittent LC Stimulation
mCherry (No Opsin)
Continuous LC Stimulation
∫Dia EMG (% baseline)
150
100
50
0
15
30
45
60
Time (mins) Post-Intervention
B
Breath Frequency (% baseline)
150
100
50
0
15
30
45
60
Time (min) Post-Intervention
C
O2 Saturation (% baseline)
150
100
50
0
15
30
45
60
Time (min) Post-Intervention

Supplementary Figure 3 – Intermittent LC Stimulation vs mCherry (no opsin) control vs Continuous LC Stimulation – Diaphragm activity (A), respiratory frequency (B), and oxygen saturation (C) did not differ between rats given ChR2-LC stimulation (blue bars) vs mCherry (no opsin; white bars) vs continuous LC stimulation (purple bars) (2-way RM ANOVA, diaphragm: F=0.50579, p=0.9994, respiratory frequency: F=0.1625, p=0.9851, O_2 saturation: F=0.2490, p=0.9565). Data presented as mean + SEM.

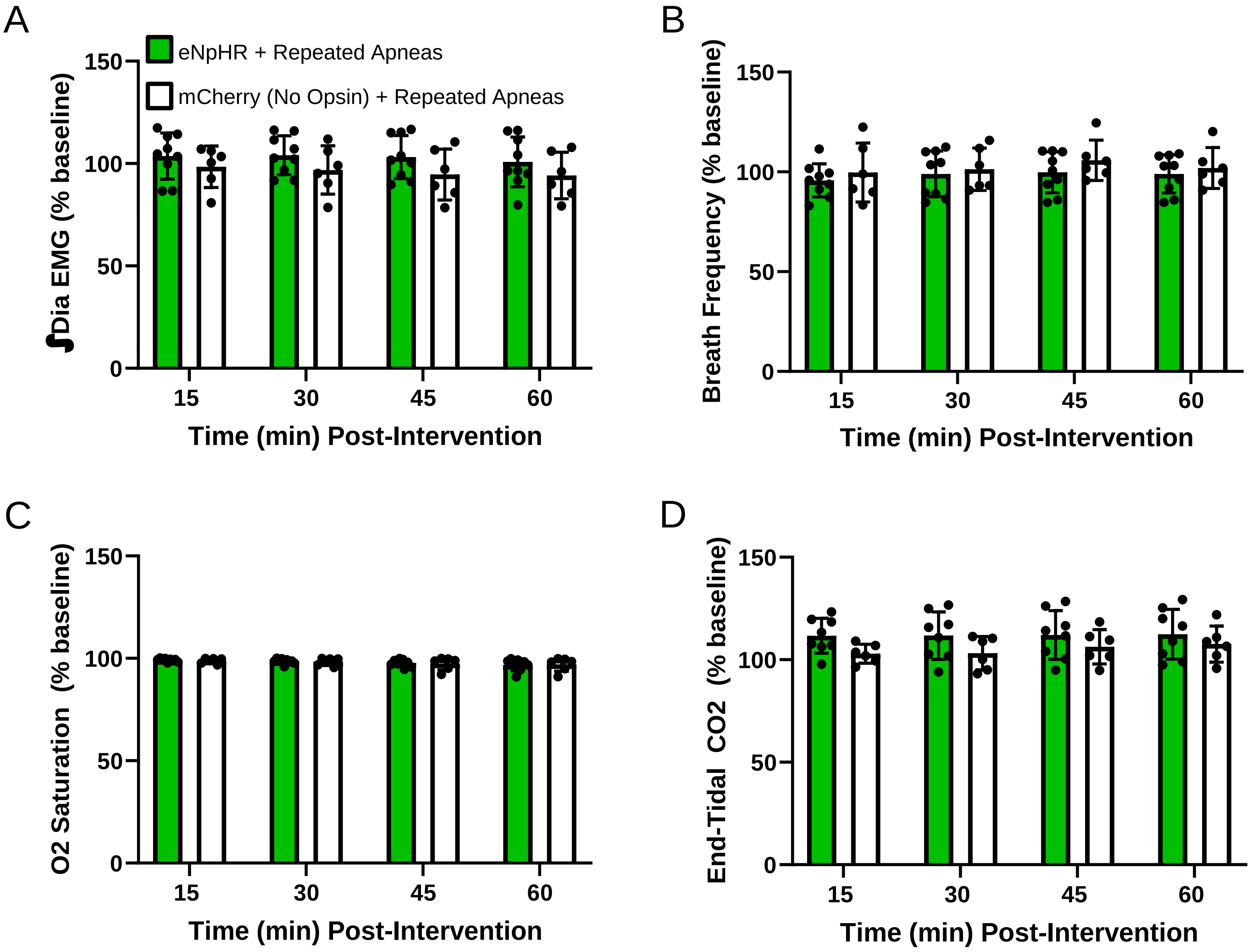
A
eNpHR + Repeated Apneas
mCherry (No Opsin) + Repeated Apneas
∫Dia EMG (% baseline)
150
100
50
0
15
30
45
60
Time (min) Post-Intervention
B
Breath Frequency (% baseline)
150
100
50
0
15
30
45
60
Time (min) Post-Intervention
C
O2 Saturation (% baseline)
150
100
50
0
15
30
45
60
Time (min) Post-Intervention
D
End-Tidal CO2 (% baseline)
150
100
50
0
15
30
45
60
Time (min) Post-Intervention

Supplementary Figure 4 – LC Inhibition + Repeated Apneas vs mCherry (no opsin) control + Repeated Apneas – Diaphragm activity (A), respiratory frequency (B), oxygen saturation (C), and end-tidal CO2 (D) did not differ between rats given eNpHR+Repeated Apneas (green bars) vs mCherry+Repeated Apneas (no opsin; white bars) (2-way RM ANOVA, diaphragm: $F=0.05135$, $p=0.9845$, respiratory frequency: $F=0.1383$, $p=0.9359$, O_2 saturation: $F=0.06316$, $p=0.9922$; end-tidal CO2: $F=0.1423$, $p=0.9341$). Data presented as mean + SEM.

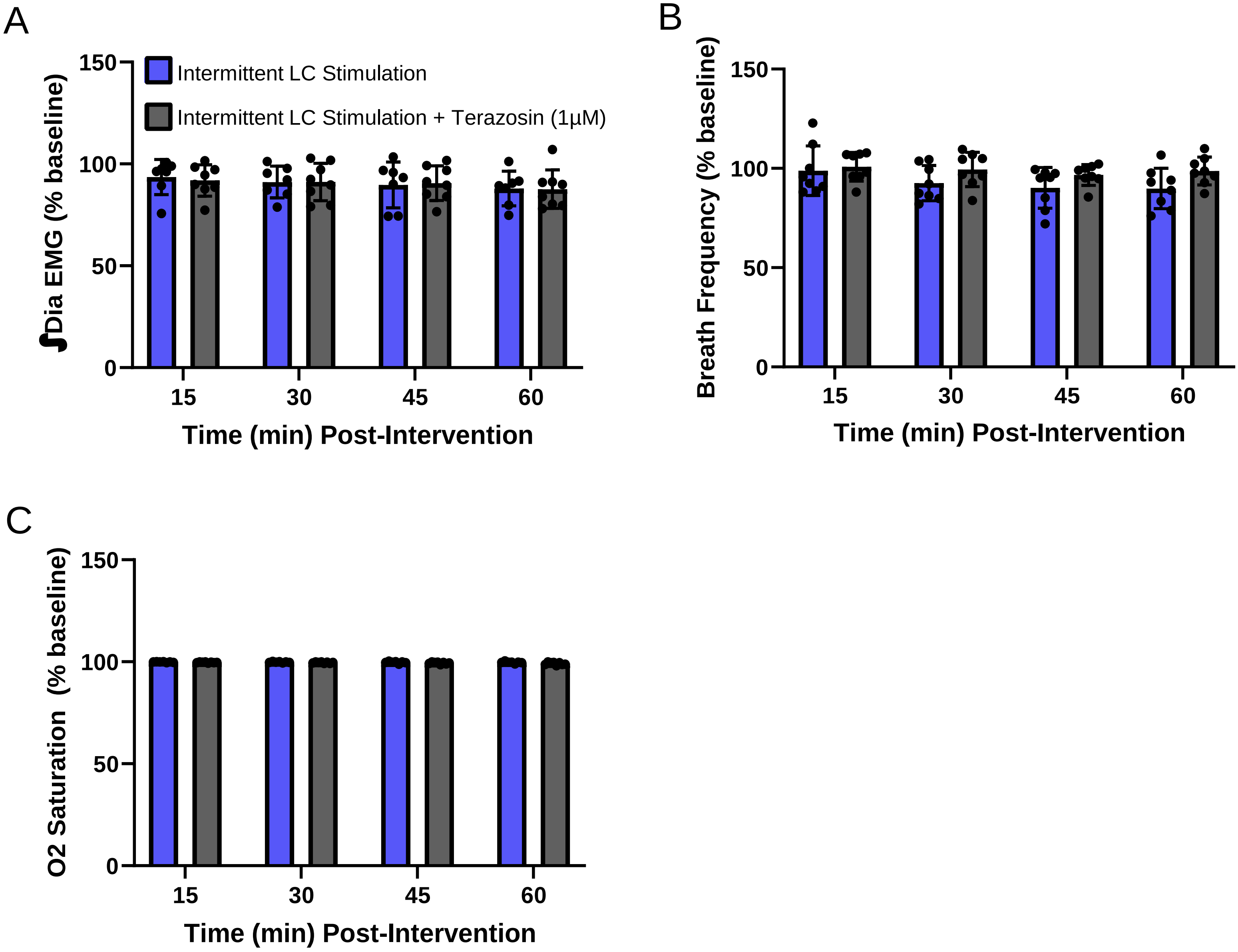
A
Intermittent LC Stimulation
Intermittent LC Stimulation + Terazosin (1μM)
∫Dia EMG (% baseline)
150
100
50
0
15
30
45
60
Time (min) Post-Intervention
B
Breath Frequency (% baseline)
150
100
50
0
15
30
45
60
Time (min) Post-Intervention
C
O2 Saturation (% baseline)
150
100
50
0
15
30
45
60
Time (min) Post-Intervention

Supplementary Figure 5 – Intermittent LC Stimulation vs Intermittent LC Stimulation + Terazosin – Diaphragm activity (A), respiratory frequency (B), and oxygen saturation (C) did not differ between rats given intermittent LC stimulation (blue bars) vs intermittent LC stimulation with terazosin (1uM) perfusion into the hypoglossal motor pool (grey bars) (2-way RM ANOVA, diaphragm: F=0.05448, p=0.9829, respiratory frequency: F=1.033, p=0.3878, O_2 saturation: F=0.6793, p=0.5733). Data presented as mean + SEM.

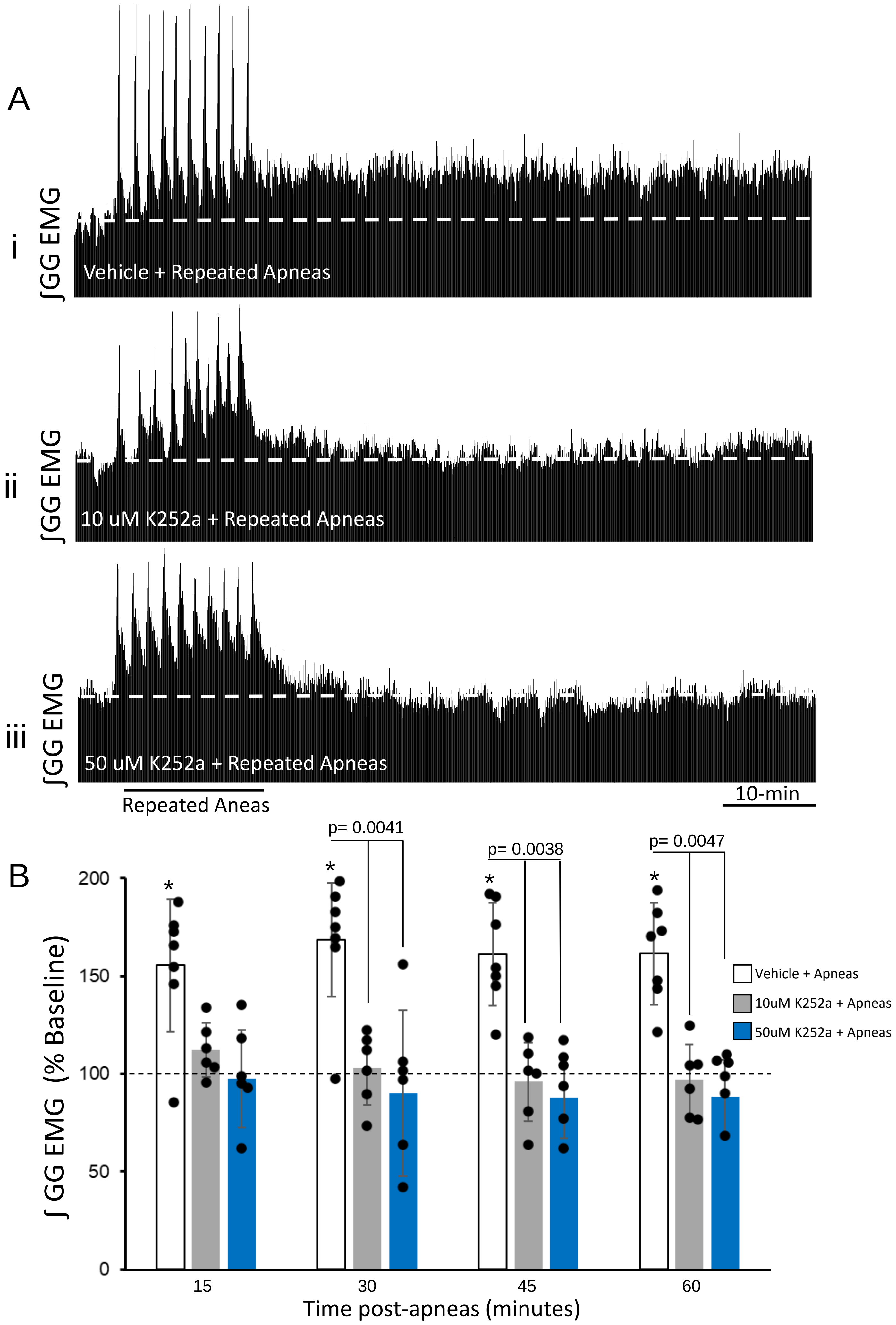

A
i
∫GG EMG
Vehicle + Repeated Apneas
ii
∫GG EMG
10 uM K252a + Repeated Apneas
iii
∫GG EMG
50 uM K252a + Repeated Apneas
Repeated Aneas
10-min
B
p= 0.0041
p= 0.0038
p= 0.0047
*
200
150
100
50
0
∫ GG EMG (% Baseline)
Vehicle + Apneas
10uM K252a + Apneas
50uM K252a + Apneas
15
30
45
60
Time post-apneas (minutes)

Supplementary Figure 6. Apnea-induced hypoglossal long-term facilitation (LTF) requires tyrosine kinase receptor signalling. (A) Representative integrated inspiratory hypoglossal EMG recordings before, during and for 60 minutes after repeated apneas. Top trace (i) demonstrates LTF of hypoglossal motor output in response to repeated apneas in a vehicle treated rat . Bottom traces (ii and iii) demonstrate that hypoglossal LTF is abolished when tyrosine kinase signaling is blocked via K252a perfusion (10uM and 50uM) at the hypoglossal motor pool before repeated apneas. (B) Mean values expressed as a percentage change from baseline (baseline = 100), showing that repeated apneas trigger LTF of hypoglossal motor output in vehicle treated rats; at 60-minutes post repeated apneas, inspiratory genioglossus EMG activity was significantly elevated above baseline levels (white bars). In separate group of rats, perfusion of either 10uM or 50uM K252a 1uM at the hypoglossal motor pool before repeated apneas abolishes the expression of hypoglossal LTF (grey and blue bars), demonstrating that apnea-induced hypoglossal LTF requires tyrosine kinase receptor signaling for its expression. Group data are expressed as a percentage change from baseline ± SD. Asterisks (*) denote a significant difference ($p<0.05$) compared to baseline at the indicated timepoint within each respective group. Indicated p values denotes a significant difference between vehicle treated rats and K252a treated rats at the indicated time points.
